# Brain functional BOLD perturbation modelling for forward fMRI and inverse mapping

**DOI:** 10.1371/journal.pone.0191266

**Published:** 2018-01-19

**Authors:** Zikuan Chen, Jennifer Robinson, Vince Calhoun

**Affiliations:** 1 The Mind Research Network and LBERI, Albuquerque, New Mexico, United States of America; 2 Department of Psychology, Auburn University, Auburn, Alabama, United States of America; 3 Auburn University MRI Research Center, Auburn University, Auburn, Alabama, United States of America; 4 University of New Mexico, Depart Electrical Computer Engineering, Albuquerque, New Mexico, United States of America; University of Maryland at College Park, UNITED STATES

## Abstract

**Purpose:**

To computationally separate dynamic brain functional BOLD responses from static background in a brain functional activity for forward fMRI signal analysis and inverse mapping.

**Methods:**

A brain functional activity is represented in terms of magnetic source by a perturbation model: χ = χ_0_ +δχ, with δχ for BOLD magnetic perturbations and χ_0_ for background. A brain fMRI experiment produces a timeseries of complex-valued images (T2* images), whereby we extract the BOLD phase signals (denoted by δP) by a complex division. By solving an inverse problem, we reconstruct the BOLD δχ dataset from the δP dataset, and the brain χ distribution from a (unwrapped) T2* phase image. Given a 4D dataset of task BOLD fMRI, we implement brain functional mapping by temporal correlation analysis.

**Results:**

Through a high-field (7T) and high-resolution (0.5mm in plane) task fMRI experiment, we demonstrated in detail the BOLD perturbation model for fMRI phase signal separation (*P* + δ*P*) and reconstructing intrinsic brain magnetic source (χ and δχ). We also provided to a low-field (3T) and low-resolution (2mm) task fMRI experiment in support of single-subject fMRI study. Our experiments show that the δχ-depicted functional map reveals bidirectional BOLD χ perturbations during the task performance.

**Conclusions:**

The BOLD perturbation model allows us to separate fMRI phase signal (by complex division) and to perform inverse mapping for pure BOLD δχ reconstruction for intrinsic functional χ mapping. The full brain χ reconstruction (from unwrapped fMRI phase) provides a new brain tissue image that allows to scrutinize the brain tissue idiosyncrasy for the pure BOLD δχ response through an automatic function/structure co-localization.

## Introduction

The blood oxygenation level-dependent (BOLD) signal [1–4] has been widely leveraged for neuroimaging studies using functional magnetic resonance imaging (fMRI). Through the use of a gradient-recalled echo (GRE) echo planar imaging (EPI) sequence and quadrature detection, the data acquisition of BOLD fMRI produces a timeseries of T2*-dephasing images, which are complex-valued in nature. Conventionally, we depict the brain functional map from the 4D T2* magnitude dataset, while discarding the 4D T2* phase dataset. However, the T2* phase conveys information concerning brain magnetic state that is different from the T2* magnitude [5,6]. Especially, the inverse mapping for brain magnetic source reconstruction can only be achieved via T2* phase rather than T2* magnitude [7–10]. The inverse mapping for BOLD fMRI seeks the dynamic BOLD magnetic source from the timeseries of T2* phase images [8].

A complex T2* image is formed by an intravoxel spin dephasing mechanism [6,8]. The image contrast is attributed to the origin of brain tissue heterogeneity in terms of inhomogeneous magnetic susceptibility property distribution that causes an inhomogeneous fieldmap via a tissue magnetization process in a main field B_0_. For BOLD fMRI studies focusing on the brain, we are concerned with the original magnetism expression (in terms of intrinsic tissue magnetic property, prior to magnetization and other MRI transformations) of BOLD information conveyed in the T2* magnitude and phase datasets. A T2* image contains morphologic distortions and is nonlinearly related to the magnetic source [5,7,8]. Consequently, the timeseries of T2* magnitude or phase images have a complicated relationship to the original BOLD responses. These relationships have been numerically simulated [5,11–15]. Even so, the BOLD fMRI model remains incomplete, in particular in the magnetic expression of a neurovascular-coupled physiologically driven BOLD signal. By decomposing a brain magnetic state into a static background and a dynamic perturbation, we may look into the insights of BOLD fMRI for the static brain parenchymal and dynamic functional contributions and their inverse mappings separately.

Our recent research [8,16–19] has demonstrated that the T2* phase data can be used to reconstruct the original brain magnetic susceptibility source (denoted by χ). We propose using a BOLD perturbation model to describe the brain χ source in a decomposition of static brain parenchyma (denoted by χ_0_) and dynamic BOLD perturbation (denoted by δχ). With dynamic/static source separation, the dataflow during T2* imaging in an accompaniment of BOLD field perturbation and BOLD phase perturbation models can be shown. Our ultimate goal is to reconstruct the BOLD δχ dataset from the T2* phase dataset by solving an inverse imaging problem [20], achieving χ-depicted intrinsic brain functional mapping.

The fMRI signals are noisy and small (accounting for less than a 5% change in energy consumption [21,22]) such that a brain functional activity is indiscernible in a snapshot of brain imaging. For a task-evoked BOLD fMRI experiment, a designed task paradigm that consists of a repetition of stimuli is always used to deal with the BOLD signal weakness. While multi-subject population statistics are widely accepted for group-level BOLD fMRI studies, a research trend of using individual fMRI study has recently emerged under the claim that “data from a single subject are actually meaningful and reliable” [23,24]. Accordingly, we applied our BOLD perturbation model to two single-subject task fMRI experimental data analyses (one 7T high-resolution dataset and another 3T low-resolution one). Without group averaging, the single-subject experimental data analysis allows us 1) to examine the brain BOLD perturbation model in technical detail; 2) to observe the single subject’s function idiosyncrasy; and 3) to co-localize a brain function map with its brain tissue structure (background) *per se*.

## Theory, model and methods

**Fig 1:**
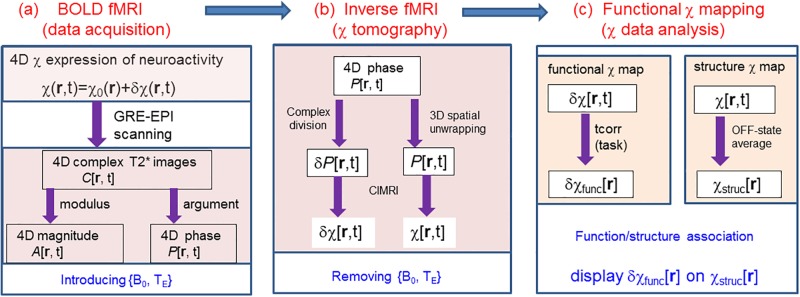
Overview diagram of BOLD perturbation model for brain imaging and functional mapping.

The overview of BOLD fMRI and its inverse for brain functional χ mapping is shown in Fig 1, which consists of a cascade of three stages: (a) BOLD fMRI for data acquisition (T2* phase imaging); (b) Inverse mappings for δχ and χ source reconstructions; and (c) brain function/structure depictions in reconstructed δχ and χ source dataspaces.

The magnetic source of BOLD fMRI is a dynamic spatiotemporal distribution of brain tissue magnetic susceptibility (primarily the change in cerebral vascular blood magnetism), denoted by χ(**r**,t). Under MRI scanning (in a static field B_0_) through the use of a GRE EPI sequence (with an echo time T_E_), the BOLD fMRI data acquisition produces a timeseries of complex-valued T2* images, denoted by C[**r**,t]. With an additive BOLD perturbation model for the magnetic source χ(**r**,t), denoted by χ(**r**,t) = χ_0_(**r**) + δχ(**r**,t), we may extract the BOLD contribution in the T2* signal (in terms of phase changes δ*P*[**r**,t], see below) and retrospectively reconstruct the BOLD magnetic source (in terms of δχ[**r**,t]) by performing inverse mapping. The ultimate goal is to depict brain functions in the reconstructed BOLD magnetic source dataspace (δχ[**r**,t]). It is noted in Fig 1 that the BOLD fMRI data acquisition introduces the MRI parameters (such as B_0_ and T_E_) that are thereafter removed by inverse fMRI, and that the BOLD perturbation modeling enables both δχ[**r**,t] and χ[**r**,t] reconstructions. More details about this technique are addressed in the followings.

### BOLD perturbation modeling for fMRI data acquisition

Let χ_0_(**r**) denote a static brain tissue magnetic state, δχ(**r**,t), which is a dynamic brain BOLD activity in an expression of cerebral blood magnetism. We assume an additive perturbation model to describe the spatiotemporal evolution of brain χ states by

$$\begin{array}{ll} \chi (r,t)=\chi_{0}(r)+\delta \chi (r,t) & \left(\chi \;\text{perturbation}\right)\\ \text{with}\;|\delta \chi |<<|\chi_{0}| & \end{array}$$ (1)

The BOLD χ perturbation model in Eq (1) greatly simplifies the complicated neurovascular process associated with a brain biophysiological activity and is very useful for numerical BOLD fMRI simulations [5,7,14,15]. Herewith, we make use of the BOLD χ perturbation model presented in Eq (1) to trace the data flow of BOLD δχ source during BOLD fMRI data acquisition.

Placed in a main field B_0_, the brain tissue is subject to a magnetization (magnetic polarization) process that manifests as a spatial orientation alignment of the magnetic dipole moments along B_0_ (parallel for paramagnetic magnetization and antiparallel for diamagnetic magnetization). Since brain tissue is a nonmagnetic material (strictly, a weak magnetic material, with |χ| ~ 1×10^−6^ in dimensionless unit in SI metric), the tissue magnetization can be linearly approximated [25] by

$$\begin{array}{l} \mu_{0}M(r)=\chi (r)B_{0}\;(\text{linear}\;\text{magnetization})\\ \text{with}\;M(r)\equiv \frac{\sum_{r\prime \in d^{3}r}m(r\prime )}{\left|d^{3}r\right|} \end{array}$$ (2)

where **m**(**r**) denotes a microscopic magnetic dipole moment, **M**(**r**) a macroscopic magnetization vector per macroscopic volume, |d^3^**r**| a volume element, and μ_0_ = 4π×10^−7^ N/A^2^, a magnetic constant (vaccum permeability). Under linear magnetization approximations, the χ-induced fieldmap is given by

$$\begin{array}{l} b(r,t)=B_{\text{0}}\chi (r,t)*h_{dipole}(r)\;\;\;\;\;\;\;\;\;\;(\text{linear}\;\text{magnetization}\;\text{approx})\\ \text{with}\;h_{\text{dipole}}(r)=\frac{1}{4\pi }\frac{3z^{2}-r^{2}}{|r{|}^{5}}\;\;\;\;\;\;\;\;\;\;\;\;\;\;\;\;\;\;\;\;\;\;\;\;(\text{dipole}\;\text{kernel}) \end{array}$$ (3)

where *h*_diople_ denotes the magnetic field distribution of a point magnetic dipole and * denotes a 3D spatial convolution. Strictly viewed, *b***(r**,t) only represents the z-component of the 3D vector field (related to the 3D magnetization vector **M**) at a time point *t*. Since the spatial convolution is a linear spatial transformation, the fieldmap resulting from χ(**r**,t) is given by

$$\begin{array}{l} b(r,t)=b_{\text{0}}(r)+\delta b(r,t)\;\;\;\;\;\;\;\;\;\;\;\;\;\;\;\;\;\;\;\;(\text{fieldmap}\;\text{perturbation})\\ \text{with}\;b_{\text{0}}(r)=B_{\text{0}}\chi_{0}(r)*h_{dipole}(r)\;\;\;\;\;\;\;\;\;\;(\text{static}\;\text{background})\\ \;\;\;\;\;\;\;\;\;\;\delta b(r,t)=B_{0}\delta \chi (r,t)*h_{dipole}(r)\;\;\;\;\;\;\;\;\;\;\;\;(\text{BOLD}\;\text{portion}) \end{array}$$ (4)

That is, the intracranial fieldmap *b*(**r**,t) consists of two parts: the static field background *b*_0_(**r**) and the dynamic BOLD field perturbation δ*b*(**r**,t). It is mentioned that *b*(**r**,t) is linearly related to χ(**r**,t), so is δ*b*(**r**,t) to δχ(**r**,t) due to the additive decomposition.

The T2*MRI detection on a snapshot of the fieldmap *b*(**r**,t) produces a complex-valued image (called a complex T2* image) by an intravoxel dephasing formula [5,15,26], as given by

$$\begin{array}{ll} C[r,t] & =\frac{1}{\left|\Omega \right|}\sum_{r\prime \in \Omega (r)}e^{i\gamma \cdot T_{E}\cdot b(r\prime ,t)}\;(\text{intravoxel}\;\text{dephasing}\;\text{mechansim})\\ & ≜A[r,t]e^{i\Phi [r,t]}\;\;\;\;(\text{complex}\;\text{in}\;\{\text{magnitude},\;\text{phase}\}\text{format}) \end{array}$$ (5)

where γ denotes the proton gyromagnetic ratio (a constant), Ω(**r**) a small voxel at **r** = (*x*,*y*,*z*), |Ω(**r**)| the voxel size (in a measure of a number of proton spins in a voxel space),and 1/|Ω| for signal normalization (*C*[T_E_ = 0] = 1). The intravoxel dephasing signal formation implements a data mapping from a real-valued continuous fieldmap *b*(**r**,t) to a complex-valued discrete T2* image *C*[**r**,t], where (**r**,t) denotes the continuous space and time variables and [**r**,t] their discrete versions. The T2* imaging is a continuous-to-discrete non-linear data transformation, which denies a closed form for an explicit analytic expression for the voxel magnitude and phase signals (the discrete *A*[**r**,t] and *P*[**r**,t]) in relation to the continuous fieldmap *b*(**r**,t).

The nonlinearity of *A*[**r**,t] can be illustrated with its 1^st^-order approximations (reported in Appendix B3) by

$$A[r,t]\approx 1\text{+}\frac{{\left(\gamma T_{E}\right)}^{2}}{2|\Omega |}\sum_{r\prime \in \Omega (r)}{\left(b(r^{\prime },t)\right)}^{2}\;({\text{1}}^{st}-\text{order}\;\text{approx}.)$$ (6)

That is, the T2* magnitude assumes a quadratic nonlinearity under the 1^st^–order approximation. Therefore, we conclude that the T2* magnitude is an inherent nonlinear transformation of the fieldmap, which disables an inverse solution due to an irreversible nonlinearity like |±*a*| = *a* ≥ 0 (the least nonlinear condition of the 1^st^–order approximation in Appendix A2 and B3). In practice, the magnitude loss (calculated by 1—*A*[**r**,t]), which represents a non-decay signal by 0), is used in fMRI instead.

Meanwhile, with 1^st^- and 2^nd^-order T2* phase signal approximations (reported in Appendix B4) and fieldmap perturbation model (in Eq (4)), we illustrate the *P*[**r**,t] nonlinearity and its perturbation decomposition by

$$\begin{array}{l} \begin{array}{ll} P[r,t] & \approx \arctan\left(\frac{\gamma T_{E}b[r,t]}{1-{\left(\gamma T_{E}\right)}^{2}b^{2}[r,t]/2}\right)\\ & \approx \gamma T_{E}b[r,t]\\ & =P_{\text{0}}\text{[}r\text{]+}\delta \Phi \text{[}r,\text{t}] \end{array}\begin{array}{c} {(2}^{\text{nd}}-\text{order}\;\text{approx}.)\\ {(1}^{st}-\text{order}\;\text{approx}.)\\ \left(\text{phase}\;\text{perturbation}\right) \end{array}\\ \begin{array}{ll} \text{with}\;P_{0}[r] & =\gamma T_{E}b_{0}[r],\\ \;\;\;\;\;\;\;\;\;\;\;b_{0}[r] & =\frac{1}{\left|\Omega \right|}\sum_{r'\in \Omega (r)}b_{0}(r^{\prime })\text{,}\\ \;\;\;\;\;\;\;\;\;\;\;\;\;b[r] & =\frac{1}{\left|\Omega \right|}\sum_{r'\in \Omega (r)}b(r^{\prime })\text{,} \end{array}\begin{array}{ll} \delta P[r,t] & =\gamma T_{E}\delta b[r,t]\\ \;\delta b[r,t] & =\frac{1}{\left|\Omega \right|}\sum_{r'\in \Omega (r)}\delta b(r^{\prime },t)\\ b^{2}[r,t] & =\frac{1}{\left|\Omega \right|}\sum_{r'\in \Omega (r)}b^{2}(r^{\prime },t) \end{array} \end{array}$$ (7)

It is shown that the T2* phase signal is nonlinearly related to the fieldmap in a general setting. The linear approximation (see Appendix) leads to a linear mapping between *b*[**r**,t] and *P*[**r**,t], and the BOLD perturbation modeling (in terms of χ = χ_0_ + δχ and *b* = *b*_0_ + δ*b*) leads to a phase perturbation decomposition (*P* = *P*_0_ + δ*P*).

### Inverse fMRI

In the context of medical imaging, the source can be reproduced by seeking an inverse imaging solution. As indicated in Eq (6), the T2* magnitude imaging is irreversible, implying that the χ source cannot be reconstructed from T2* magnitude images. Fortunately, we can perform an inverse solution to the linear T2* phase imaging model in Eq (7). In corresponding to two forward mappings (χ(**r**,t)→ *b*(**r**,t)→ *P*[**r**,t]), we perform two inverse mappings (*P*[**r**,t] → *b*[**r**,t] → χ[**r**,t]) by a computed inverse MRI (CIMRI) model [20]. The BOLD perturbation model offers a two-step forward mapping δχ(**r**,t) → δ*b*(**r**,t) → δ*P*[**r**,t] for a continuous-to-discrete conversion and a two-step inverse mapping δ*P*(**r**,t) → δ*b*[**r**,t] → δχ[**r**,t] for a discrete source reproduction.

### Brain χ[r,t] source reconstruction

A raw T2* phase image is usually severely wrapped, especially for high-field T2* phase imaging. The phase wrapping phenomenon can be removed through the use of a phase unwrapping algorithm. There have been many reports on 3D MRI phase unwrapping. Perhaps the most efficient 3D phase unwrapping can be achieved by a Laplacian technique [27,28], which has been used for brain phase image processing [16,29]. Applied to a 3D wrapped phase image *P*^*wrap*^(**r**) ∈ [-π, π) rad, the Laplacian unwrapping algorithm is expressed [16,29] by

$$\begin{array}{l} P^{unwrap}=FT^{-1}\left\{\frac{FT\left\{\cos P^{wrap}\cdot FT^{-1}\left(k^{2}\cdot FT(\sin P^{wrap})\right)-\sin P^{wrap}\cdot FT^{-1}\left(k^{2}\cdot FT(\cos P^{wrap})\right)\right\}}{k^{2}}\right\}\\ \text{with}\;P^{\text{wrap}}\in [-\pi ,\pi )\;\text{and}\;P^{unwrap}\in (-\infty ,\infty ) \end{array}(\text{unwrapping})$$ (8)

where *FT* and *FT*^-1^ is a Fourier transform pair (with an identity *FT*^-1^(*FT*) = 1) and *k* = |**k**| represents the 3D discrete coordinates in the 3D Fourier domain. Besides phase unwrapping, the Laplacian algorithm in Eq (8) can largely remove the harmonic background phase [16,29] (in principle of ∇^2^e^i*P*(**r**)^ = 0). By applying a 3D Laplacian unwrapping procedure to each 3D phase image in *P*^*wrap*^[**r**,t] at a time point t, we obtain a 4D unwrapped phase dataset *P*^*unwrap*^[**r**,t] ∈ (-∞,∞) rad.

With the linear T2* phase imaging model in Eq (7), from an unwrapped phase image (denoted by *P for succinct*), we can reconstruct the fieldmap by

$$b[r,t]=\frac{P[r,t]}{\gamma T_{E}}\;(\text{linear}\;\text{T}2*\;\text{phase}\;\text{imaging}).$$ (9)

It is noted that *b*[**r**,t] and *P*[**r**,t] are spatially conformed to the scale difference (1/(γT_E_)) at each snapshot time *t*. The linear inverse mapping from *P*[**r**,t] and *b*[**r**,t] cancels the T_E_ parameter dependence.

Using the *b*[**r**,t] dataset, we can then reconstruct a χ[**r**,t] dataset by solving a dipole inversion problem. An iterative solution is given by

$$\chi [r,t]=\underset{\chi [r,t]}{\text{arg}\;\text{min}}\left(B_{0}\chi [r,t]*h_{dipole}[r]-b[r,t]\right).$$ (10)

where the reconstructed 4D χ[**r**,t] provides a discrete representation of the continuous brain dynamic source χ(**r**,t), hence the 4D χ tomography [30,31]. It is noted that the parameter B_0_ is canceled and the spatial convolution is removed in Eq (10). The discreteness effect associated with the computationally tomographic χ reconstruction diminishes as the spatial resolution increases.

The solution to the iterative dipole inversion problem is a nontrivial task. We refer readers to different solutions, such as truncated inverse filtering (filter truncation regularization) [32,33], iterative *L*_1_-norm or *L*_2_-norm regularizations [34–38], and total-variation (TV)-regularized split Bregman iteration (TVB) [20,30,34,39,40].

### BOLD δχ source reconstruction

From a 4D T2* phase dataset, we can extract the dynamic relative phase change (relative to a reference or baseline) by a complex division algorithm [8]:

$$\begin{array}{l} \delta P[r,t_{n}]=Arg\left(\frac{\text{exp}\left(iP[r,t_{n}]\right)}{\text{exp}\left(iP_{ref}[r]\right)}\right)\text{,}\;n=[1,2,\cdots ,N_{t}]\\ with\;P_{ref}[r]=P[r,t_{1}] \end{array}$$ (11)

where *Arg* denotes an operator to find the phase angle (or argument) of a complex number (via a trigonometric function *arctan*) and *N*_t_ is the number of snapshot captures in a 4D T2* dataset. The reference phase image *P*_ref_[**r**] is selected from a T2* phase image captured at a time point (not necessarily the 1^st^ time point). The complex division serves as a phase subtraction algorithm to find the phase difference between two phase images. Notably, it can correctly extract the relative phase change δ*P*[**r**,t] between two wrapped phase images as long as |δ*P*| < π. For a timeseries of severely wrapped T2* phase images, we can extract the temporal phase changes (relative to a snapshot reference) using the complex division in Eq (11), obtaining a 4D dataset δ*P*[**r**,t] in which each 3D δ*P* map is free from phase wrapping phenomenon (|δ*P*| < π). In this sense, the complex division algorithm implements phase unwrapping in the time domain [8,9]. Obviously, the static phase background *P*_0_(**r**) (the culprit of phase wrapping effect) is completely removed by the complex division in Eq (11).

From the 4D dataset δ*P*[**r**,t], we can reconstruct a 4D dataset δχ[**r**,t] in the same manner as for 4D χ[**r**,t] reconstruction, as represented by

$$\delta \chi^{recon}[r,t]=\underset{\delta \chi [r,t]}{\text{arg}\;\text{min}}\left(B_{0}\delta \chi [r,t]*h_{dipole}[r]-\frac{1}{\gamma T_{E}}\delta P[r,t]\right)$$ (12)

Again, the MRI parameters {B_0_, T_E_} are cancelled and the dipole effect is removed in the iterative minimization solution.

### Brain function map extraction

As discussed above, a brain activity may be represented in a variety of timeseries images from different perspectives, such as in T2* image dataspaces by {‘*A*[**r**,t]’, ‘*P*[**r**,t]’, ‘δ*P*[**r**,t]’} and in source dataspaces by {‘χ[**r**,t]’, ‘δχ[**r**,t]’}. Since the BOLD response is very weak (< 5%), a measurement repetition through a designed paradigm is needed for fMRI data acquisition and a statistic parametric mapping (SPM) technique is need for brain function map (*fmap*) extraction. In particular, we are interested in the brain functional mappings in the magnitude image dataspace (*A*[**r**,t]) and the reconstructed BOLD magnetic source dataspaces (χ[**r**,t] and δχ[**r**,t]).

For a 4D dataset from a task-evoked BOLD fMRI study, which acquires a 4D fMRI dataset through a designed task paradigm with a timecourse of *task*[t], we can extract the task-stimulated *fmap* by a task-correlation algorithm [16,18], as given by

$$\begin{array}{ll} \Lambda_{tcorr}[r] & =tcorr\left(\Lambda [r,t],task^{*}[t]\right),\;\text{for}\;\Lambda =\{\prime A\prime ,\prime \chi \prime ,\prime \delta \chi \prime \}\\ & \text{with}\;task^{*}[t]=task[t]*hrf[t] \end{array}$$ (13)

where *tcorr* stands for temporal correlation, which reduces the 4D spatiotemporal dataset into a 3D spatial map, and *hrf*[t] for a canonical hemodynamic response function (available in SPM software), which accounts for the lagging neurovascular response in response to a task stimulus. The 3D *tcorr* map offers an intuitive understanding of brain function pattern: a large positive *tcorr* represents a high correlative response, while a negative *tcorr* constitutes an anti-correlation response. It is pointed out that the temporal correlation data analysis on a 4D BOLD dataset can largely suppress the effect of physiological fluctuations (e.g., heartbeat and breathing cycles) as long as the task paradigm (a long timecourse of task waveform) is out of the phase with the physiological pulsations.

### Signal-to-noise ratio (SNR) and contrast-to-noise ratio (CNR)

A task-evoked BOLD fMRI experiment is essentially a time-locked weak signal repetition detection technique. Through the use of complex division, we can extract the pure BOLD responses, which are buried in heavy noise. In the experiment result report below, we provided signal-to-noise ratio (SNR) and contrast-to-noise ratio (CNR) measurements to numerically characterize the pure BOLD responses at different stages (in the original source δχ, the δχ-induced fieldmap δ*b*, and the output image δ*P*). Based on the SNR and CNR definitions reported in [41], we define the dynamic SNR and CNR metrics for a timeseries of images by

$$\begin{array}{ll} SNR[t] & =\frac{\left|mean\left(ROI_{act}[t]\right)\right|}{std\left(ROI_{inact}[t]\right)}\\ CNR[t] & =\frac{\left|mean\left(ROI_{act}[t]\right)-mean\left(ROI_{inact}[t]\right)\right|}{std\left(ROI_{inact}[t]\right)} \end{array}$$ (14)

where *mean*(·) and *std*(·) stand for statistic mean and standard deviation, ROI_act_[t] and ROI_inact_[t] for activation and inactivation regions of interest at a time point t. The ROI_act_ and ROI_inact_ can be retrospectively specified (through a visual selection of an activation blob and an inactive region in an *fmap* afterward). It is mentioned that the signal noise measurement at an air region (defined in [41]) is applicable for phase signal noise measurement because there is no water proton (signal carrier) in an air region that consequently causes capricious phase signals therein. Instead, we define an inactive tissue region (retrospectively specified) for noise measurement.

### Function/structure co-localization

Conventionally, brain anatomical structure is procured by a high-resolution T1 scan (at submillimeter resolution). The *fmap* is always displayed over a high-resolution T1 image, which can be obtained by T1 imaging on the same brain (intra-subject inter-scan data acquisition) or adopted from a standard brain template (inter-subject data). The function/structure association requires intra-subject image coregistration that is computationally intensive and prone to digital errors; and additionally, the inter-subject function/structure association suffers from a loss of the brain tissue structure individuality.

Upon availability of high-resolution GRE-EPI data and subsequent high-resolution χ source reconstruction, we propose to co-localize an *fmap* on a brain χ structure background. Since all the data (including raw data and processed data) are derived from the same source at a single scan (intra-scan data derivations), they are spatially automatically coregistrated. High field fMRI enables high-resolution functional mapping and function/structure association with rich spatial information, especially for hopefully scrutinizing the vascular origin of brain function source.

### Task fMRI experiments

We conducted one high-field high-resolution task fMRI experiment at the Auburn University MRI Research Center on a healthy adult volunteer. The human subject MRI scanning was approved by the Institutional Review Board at the Auburn University. Written consent was obtained from the human subject before the MRI scanning. The subject was instructed to perform a finger-tapping task using the scan session. The scanner was Siemens MAGNETOM 7T scanner and we used a standard GRE-EPI sequence with the following parameter settings: T_R_/T_E_ = 3000/29 ms, flip angle = 70°, slice spacing = 0mm, slice oblique = 0°, voxel size = 0.5 × 0.5 × 1.2 mm^3^, and matrix = 234 × 234 × 24 voxels for a coverage of the superior-most portion of the brain encompassing the motor cortex (slab thickness = 28.8mm). These complex images were reconstructed from 32 coil elements through a GRAPPA PAT mode with an acceleration factor of 3 in the phase-encoding dimension and a sum-of-square algorithm for coil combination (which is optimal for magnitude MRI but suboptimal for phase MRI, leaving a room for improvement). The task consisted of 15-second blocks alternating between task and rest for a total of 50 volumes (5 cycles of {5 ON, 5 OFF}). An in-house modification to the acquisition protocol allowed for the production of both 4D raw magnitude and 4D phase datasets as denoted by *A*[*x*,*y*,*z*,*t*] and Φ[*x*,*y*,*z*,*t*], in a matrix form of 234×234×24×50.

We also conducted a low-field low-resolution finger tapping task fMRI experiment by scanning a different healthy adult volunteer in a Siemens 3T TrioTim scanner at the Mind Research Network (MRN). The subject experiment was approved by the Institutional Review Board at MRN and by the informed written consent from the subject. The task paradigm was designed as a block timecourse consisting of 5 cycles of {15 OFF, 15 ON} plus 15 OFF, a total number of 165 timepoints. With the experiment settings {standard GRE-EPI sequence, T_R_/T_E_ = 3000/29ms, flip angle = 75°, isotropic voxel = 2 × 2 × 2 mm^3^, matrix = 128 ×128×30 voxels for a brain superior coverage}, we obtained a pair of 4D magnitude and phase images in a matrix form of 128 ×128 ×30 ×165.

## Results

**Fig 2:**
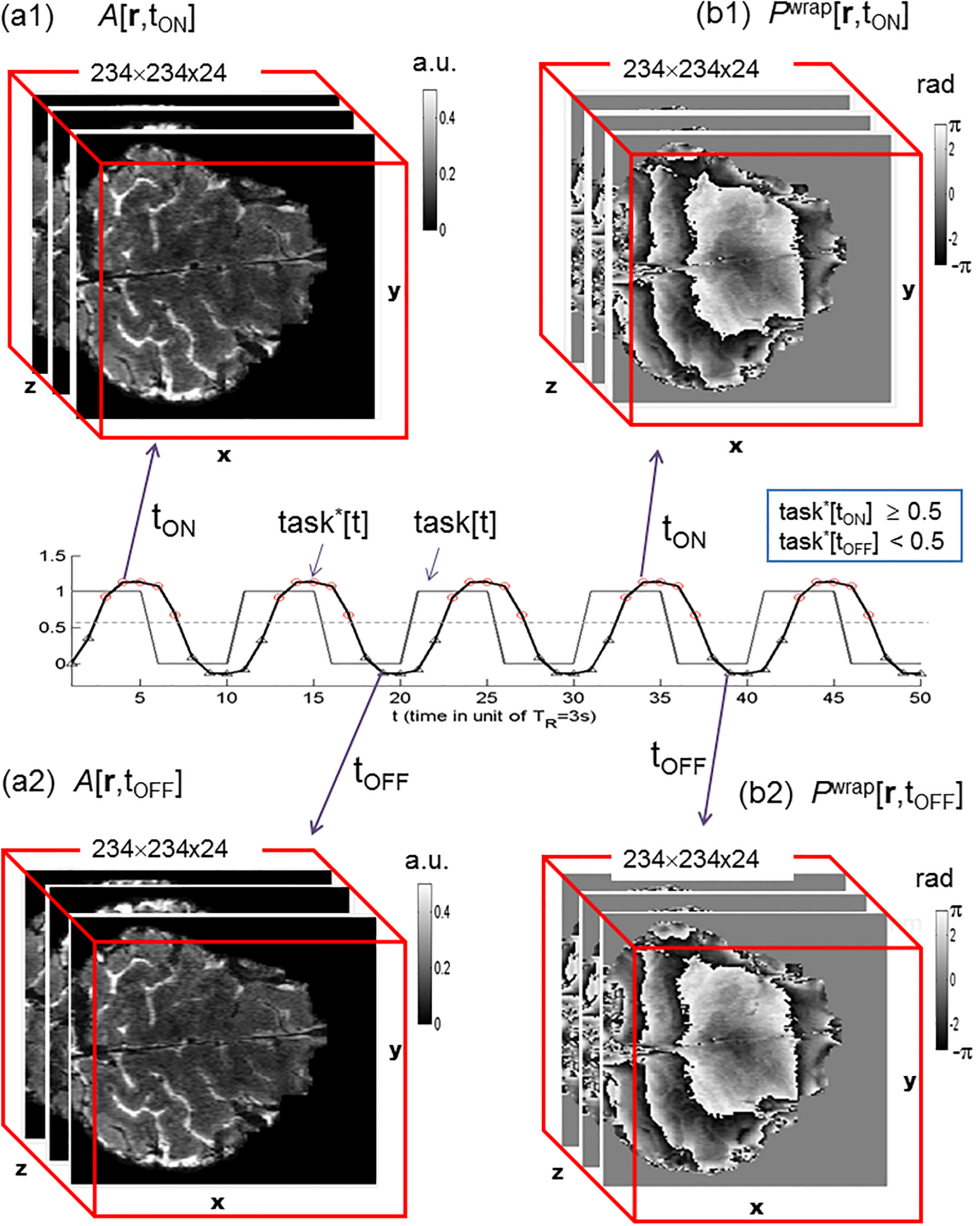
T2* magnitude and phase image acquisition under a task paradigm. The ON states are defined by task*[t] ≥ 0.5 and the OFF states by task*[t] < 0.5.

In this section, we report our experimental results by applying our BOLD perturbation model to two single-subject task fMRI data analyses (data acquisition method was described in the previous section). We applied our perturbation model to these two task fMRI datasets separately by performing forward fMRI phase perturbation decomposition and inverse mapping through the same procedure. We reported the high-resolution 7T task fMRI experimental results in Figs 2 through 6 in technical detail, while summarized the low-resolution 3T task fMRI experimental results in Fig 7.

**Fig 3:**
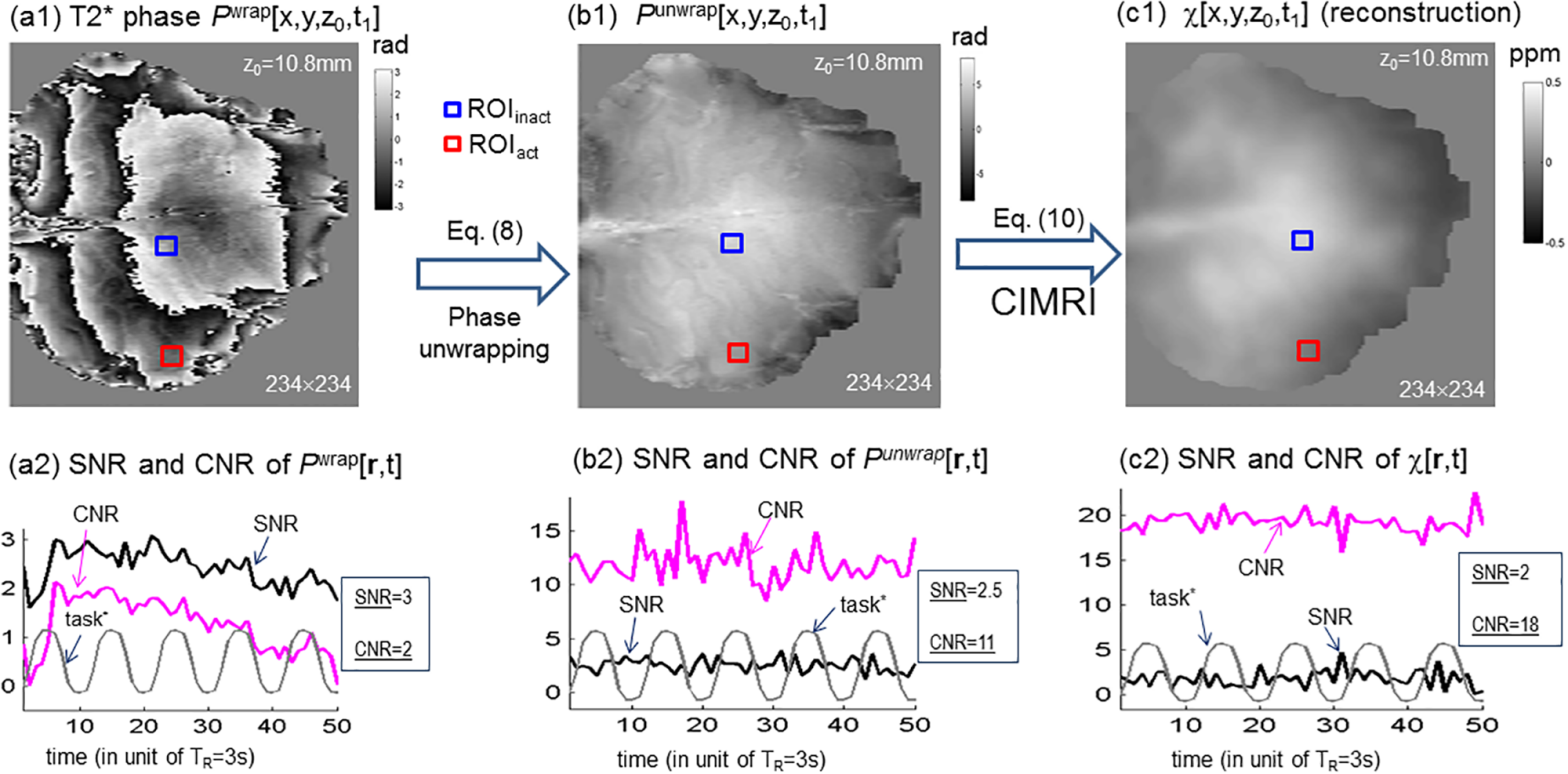
(a1,b1,c1) T2* phase processing and χ[r,t] reconstruction; (a2,b2,c2) SNR and CNR characterizations. The SNR CNR values were calculated according to the definition in Eq (14), with the ROI_act_ and ROI_inact_ defined retrospectively in Fig 5(c2), in a small size of 5 × 5 × 3 voxels. The task* is included to observe the dynamics of SNR and CNR with respect to the stimuli. The SNR and CNR denote time averages.

**Fig 4:**
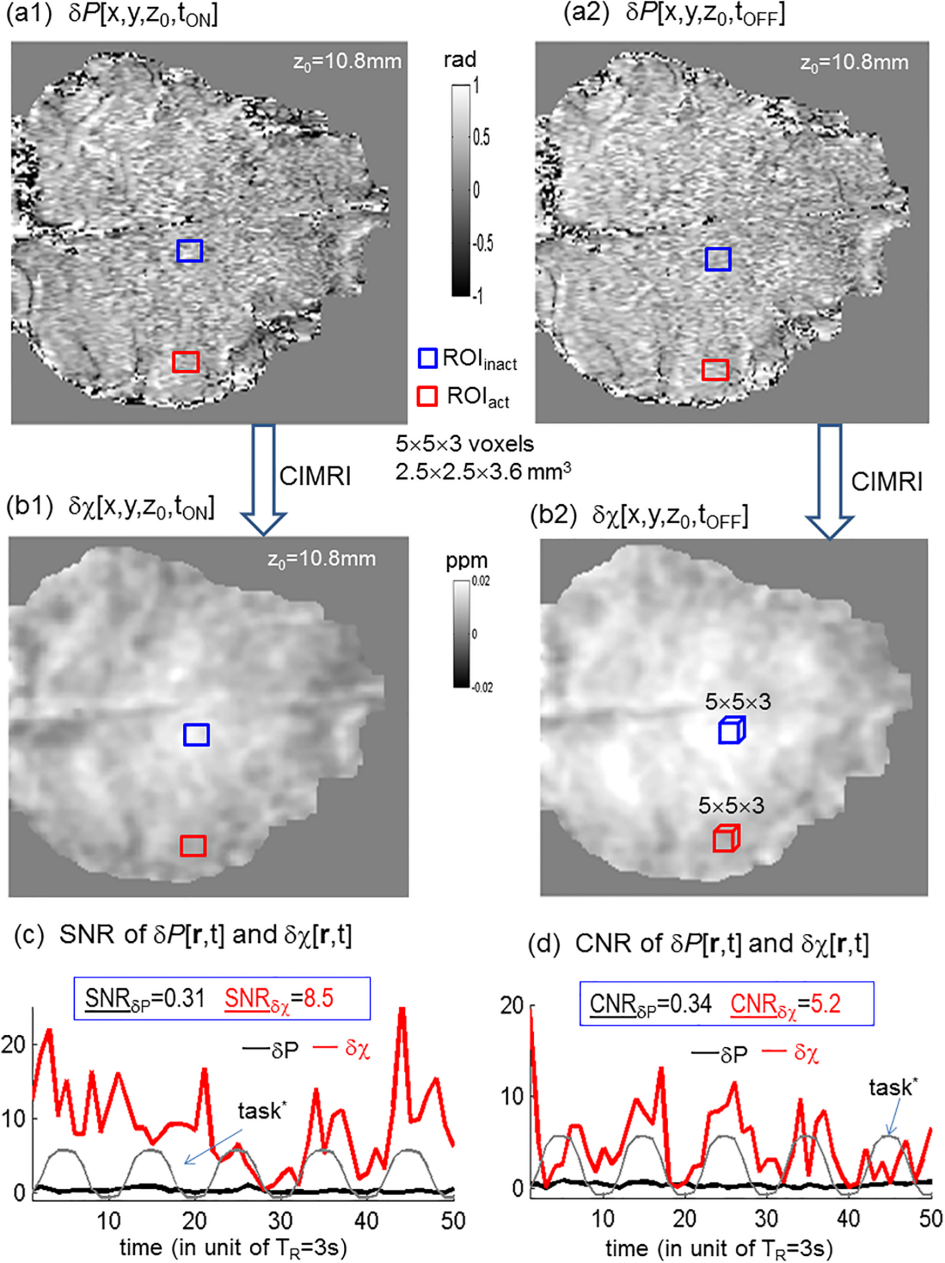
The BOLD δχ[r,t] reconstruction from δ*P*[r,t] by CIMRI (demonstrated with a z_0_ slice in a volume at an ON and OFF snapshot). The SNR and CNR values were calculated according to the definition in Eq (14), with the ROI_act_ and ROI_inact_ defined retrospectively in Fig 5(c2), in a small size of 5 × 5 × 3 voxels. The SNR and CNR denote time averages. The task* is included to observe the dynamics of SNR and CNR with respect to the stimuli. The ON and OFF snapshots are labeled in Fig 2.

**Fig 5:**
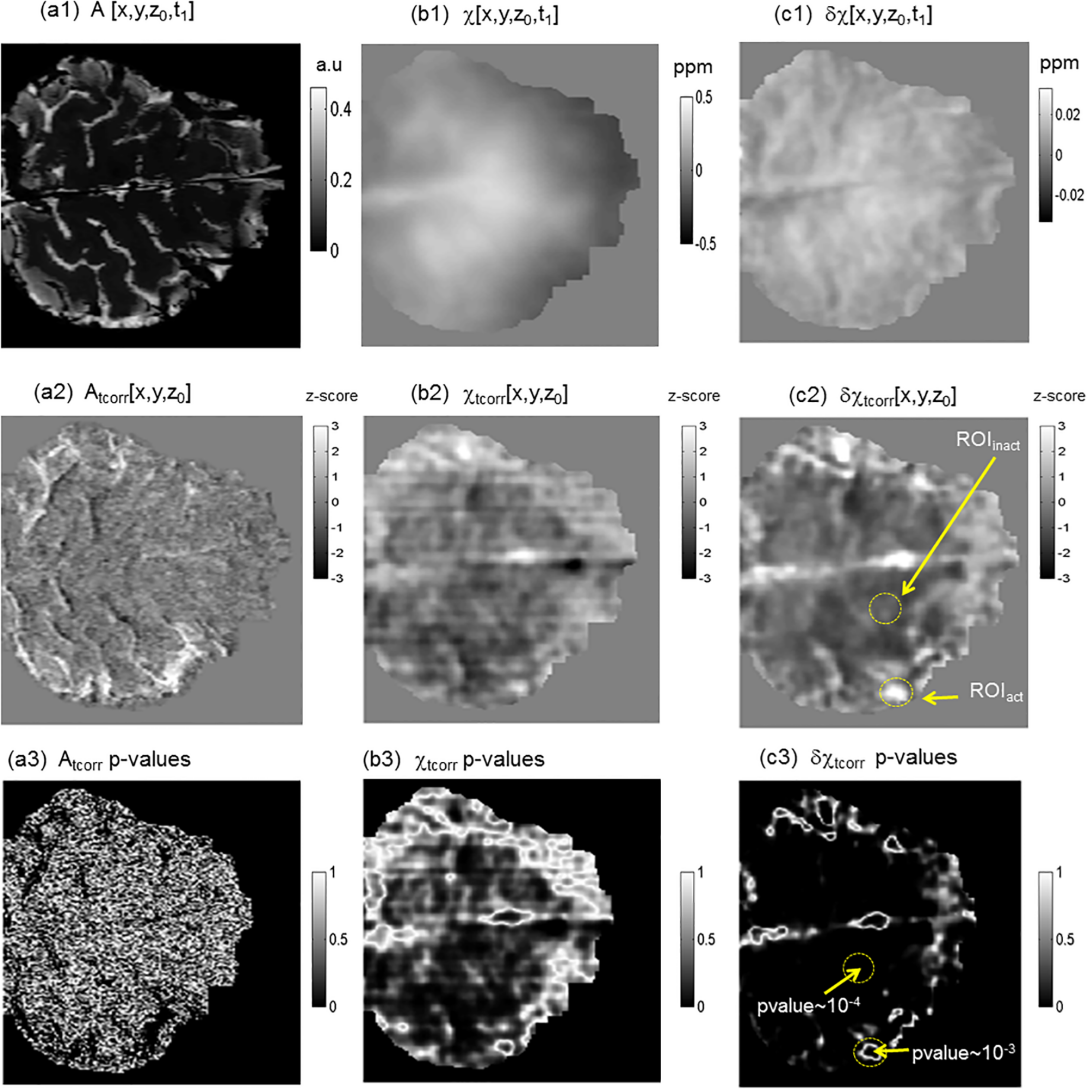
Task-evoked brain functional mappings in different dataspaces: (a1, a2, a3) T2* magnitude image dataspace *A*[r,t]; (b1,b2,b3) the reconstructed χ[r,t] source dataspace (including static background); (c1,c2,c3) the reconstructed δχ[r,t] source dataspace (pure BOLD χ responses). In (c2), the ROI_act_ defines a task-evoked active region of interest, and the ROI_inact_ defines a task-irrelevant region of interest.

**Fig 6:**
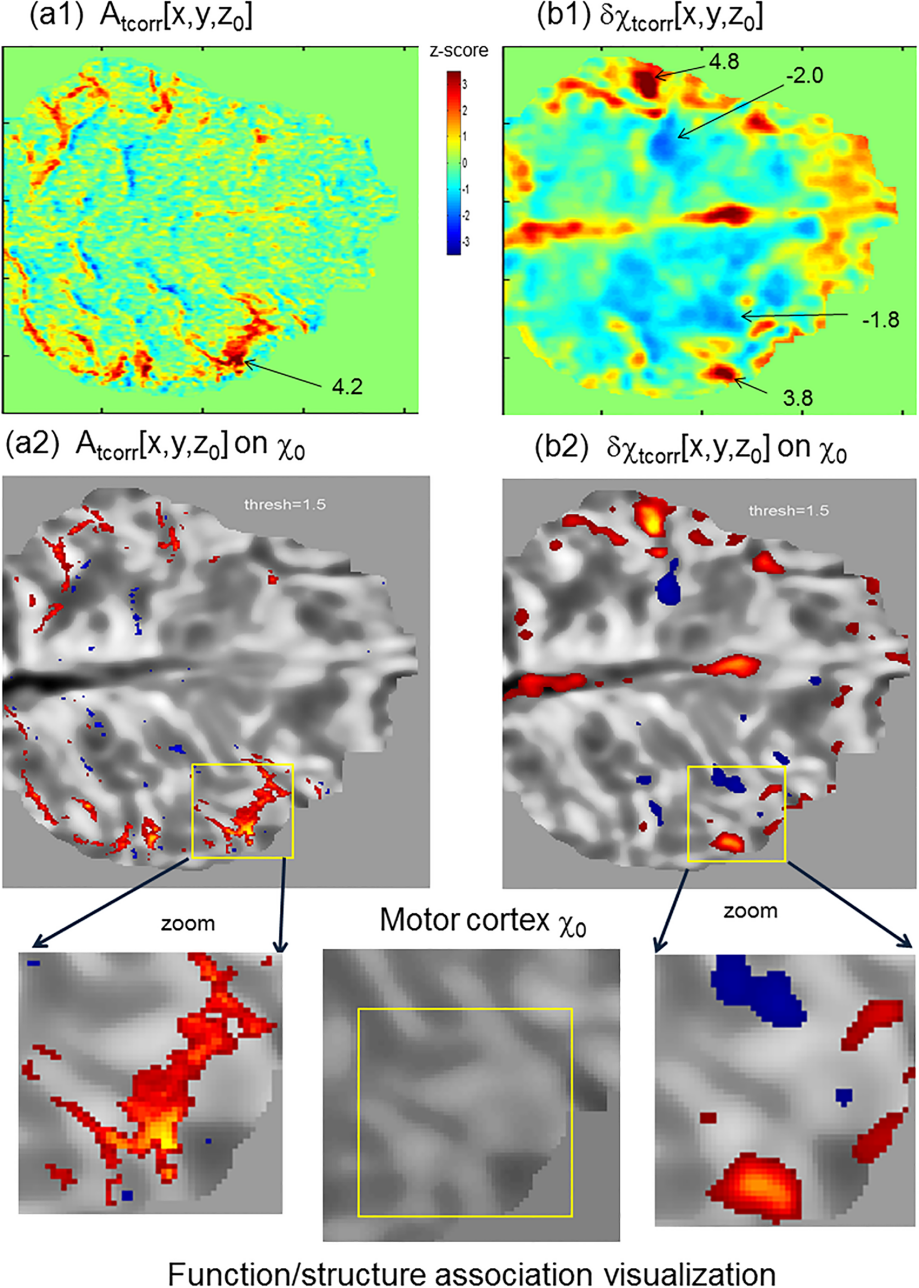
Visualization of magnitude- and susceptibility-depicted brain *fmap*s (on the reconstructed χ motor cortex image). (a1,a2) Magnitude-based *fmap* (A_tcorr_); (b1, b2) BOLD χ-depicted *fmap* (δχ_tcorr_). The magnified insets are for scrutinizing function/structure associations. The numbers in (a1,a2) denote the z-scored *tcorr* values at the activation blobs.

**Fig 7:**
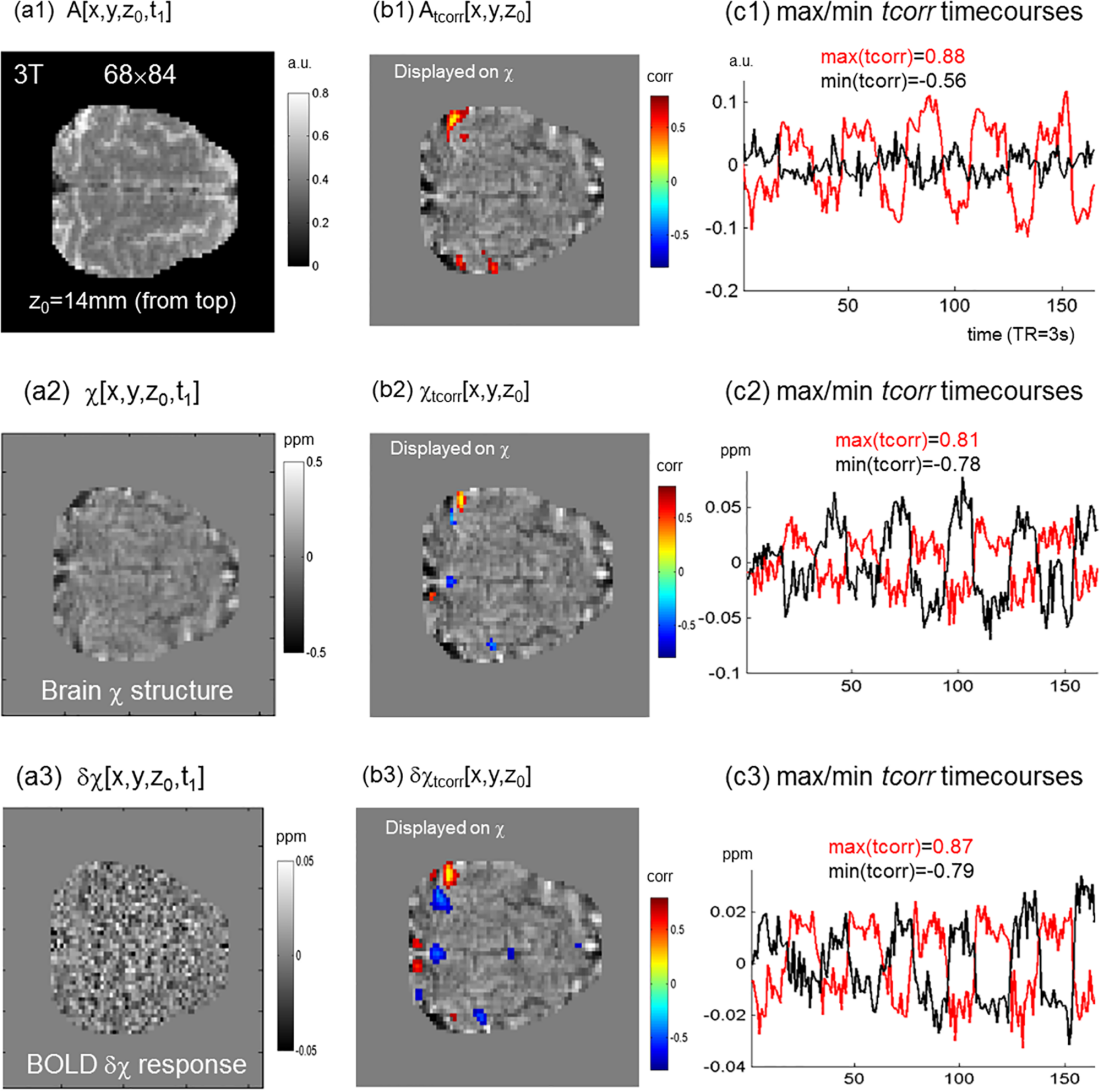
A low-field (3T) and low-resolution (2mm) brain task fMRI experiment (finger tapping) with task function analyses in different dataspaces: (a1,b1,c1) fMRI magnitude, (a2,b2,c2) reconstructed susceptibility (χ), and (a3,b3,c3) reconstructed temporal susceptibility change (δχ). A brain snapshot state (at a timepoint t_1_) was displayed with an axial slice (z_0_ = 14mm from brain top) in (a1) magnitude, (a2) reconstructed χ, and (a3) reconstructed δχ. Correspondingly, task correlation *fmap’*s were displayed in (b1,b2,b3); and the maximal (red) and minimal (black) task-correlated voxel timecourses were plotted in (c1,c2,c3). Note that the functional *tcorr* maps (b1,b2,b3) were displayed over the reconstructed brain χ image (a2). Display units: *a*.*u*., arbitrary unit (dimensionless); *corr*, correlation value in range [–1, 1] (dimensionless); *ppm*, parts per million 10^−6^ (dimensionless).

In Fig 2(a1, b1) we showed a pair of the 7T fMRI magnitude and phase images (raw data) acquired at a snapshot of task-ON state (defined by task*[t] ≥ 0.5), and in Fig 2(a2, b2) for the 3D images at a task-OFF state (defined by task*[t] ≥ 0.5). The task paradigm for capturing 50 snapshot images is also displayed in Fig 2, in which the waveform task*[t] accounts for the lagging hemodynamic responses. More slices (16 axial slices selected from a 24-slice volume) in a snapshot volume were presented in S1 and S2 Figs. It is noted that the raw T2* phase images are severely phase wrapped in the data range of [-π, -π) rad, and that the differences between a task-ON and task-OFF state (either in magnitude images (a1,a2) or in phase images (b1,b2)) are too subtle to be visually perceived.

In Fig 3(a1, b2) we illustrated the 7T fMRI phase image processing (using Eq (8)) and the χ[**r**,t] reconstruction (using Eq (10)), displayed with an axial slice (at z_0_ = 10.8 mm distant from brain top). The SNR and CNR values were calculated from the 3D images according to the definition in Eq (14), with the ROI_act_ and ROI_inact_ (in size of 5 × 5 × 3 voxels) retrospectively defined in Fig 5(c2). The task*[t] was included for observing the dynamics of SNR and CNR with respect to the stimuli. The SNR and CNR numbers denote the time averages (over *N*_t_ -1 = 49 timepoints). More z-slices of the reconstructed χ[**r**,t] (at one time point t_1_) were presented in S3 Fig.

From the phase perturbation dataset δ*P*[r,t] (obtained by the complex division in Eq (11)), we reconstructed the pure BOLD χ response dataset δχ[**r**,t] using the TVB algorithm (in Eq (12)), as demonstrated in Fig 4. More z-slices of reconstructed δχ[**r**,t] (at one time point t_1_) were presented in S4 Fig. Note that the task-evoked small BOLD response (in terms of δ*P* or δχ) was not visually discernible in a snapshot image in Fig 4.

In Fig 4, we also provided the SNR and CNR measurements for δ*P*[**r**,t] and δχ[**r**,t] datasets (calculated by Eq (14) with ROI_act_ and ROI_inact_ defined in Fig 5(c2)). We observe the following aspects: i) the reconstructed χ[**r**,t] and δχ[**r**,t] assume different value ranges: χ∈[-0.5, 0.5] ppm (part per million, 10^−6^, in SI metric) and χ∈[-0.03, 0.03] ppm, indicating the weakness of the BOLD perturbation δχ; ii) The spatial maps of χ[**r**,t] and δχ[**r**,t] are more spatially smoothed than those of P[**r**,t] and δP[**r**,t] due to the removal of the spatial derivative property of dipole effect (5); and iii) The dataset δχ[**r**,t] gains higher SNR and CNR values than the dataset δ*P*[**r**,t] does (specifically, SNR_δχ_ = 8.5 and CNR_δχ_ = 5.2 versus SNR_δP_ = 0.31 and CNR_δχ_ = 0.34).

From 4D datasets {*A*[**r**,t], χ[**r**,t] and δχ[**r**,t]} (displayed with a z-slice in Fig 5(a1,b1,c1)), we extracted the task-evoked brain *fmaps* using Eq (13), and obtained the 3D *fmap*s {*A*_tcorr_[**r**],χ_tcorr_[**r**], δχ_tcorr_[**r**]} as displayed in Fig 5(a2,b2,c2). With inspection, we could identify the brain active blobs at the motor cortex. We define an active and inactive region of interest (denoted by ROI_act_ and ROI_inact_, in size of 5 × 5× 3 voxels, corresponding to 2.5 × 2.5 × 3.6 mm^3^) for image SNR and CNR calculations (in Eq (14)). In (a3,b3,c3), we also provide the p-values for the *tcorr* calculations, which reveals an edge effect of *tcorr*-based *fmap* extractions in the reconstructed source dataspaces (in the χ_tcorr_ and δχ_tcorr_ maps). Specifically, the maximal *tcorr* was calculated at the activation foci (inside the blob in the ROI_act_) with a very small p-value (< 10^−2^ for δχ_tcorr_ in Fig 5(c3)), implying a high statistical stability for the correlation-based task idenfication. It is noted in Fig 5 that the task functional mapping using the reconstructed χ data (b1,b2,b3) is less statistically reliable than using the reconstructed δχ data (c1,c2,c3) (as inferred from the p-value maps (b2) and (c2)). The montage displays for the 3D *tcorr fmap*s are presented in S5, S6 and S7 Figs.

Finally, we need to visualize the function/structure association. Instead of adopting a brain anatomical template, we used a brain structure image (denoted by χ_0_[**r**]) for *fmap* visualization by selecting a 3D χ source volume from χ[**r**,t]. We performed image enhancement (e.g., a homodyne filtering [42]) on χ_0_[**r**] to enhance the cortex structure (gyri and sulci patterns). The montage displays of 3D image χ_0_[**r**] are presented in S8 Fig. At the 0.5 mm in-plane spatial resolution, the reconstructed χ_0_ images reveal superior sagittal sinus and cortical details (gyri and sulci patterns), but the intra-cortex vasculature remains undiscernible.

In Fig 6, we visually compare the magnitude-depicted *fmap A*_tcorr_[**r**] and the δχ-depicted *fmap* δχ_tcorr_[**r**] by color displays in (a1,a2), thresholded blob displays on reconstructed χ structure background in (a2,b2), and magnified blob displays in the insets. It is observed that the δχ_tcorr_[**r**] *fmap* reveals bidirectional BOLD χ responses to the task stimuli with compact and strong activation blobs. In comparison, the conventional *A*_tcorr_[**r**] *fmap* appears a positive dominance with more spatially spread in response patterns. Since all the images were derived from the same scan data (intra-scan data), they were automatically self-aligned, thus facilitating the function/structure associations.

In Fig 7, we showed the low-resolution (2mm) 3T task fMRI data analysis results. In the top row (a1,b1,c1), we displayed the task functional mapping in terms of the brain structural image (in an axial slice), the task-evoked *fmap* (in an axial slice) and the most positive and negative responsive timecourses (at one activation focus with *max*(*tcorr*) for task correlation and at another activation focus with a negative *min*(*tcorr*) for task anticorrelation). In the middle row (a2,b2,c2) were showed the task functional mapping using the reconstructed full χ data (BOLD response was superimposed on the background); and in the bottom row (a3,b3,c3) were showed the pure BOLD response using the reconstructed δχ data by excluding the static background.

The 3T experimental results in Fig 7 reveal the following aspects: 1) The pure BOLD δχ response was too weak and noisy to be observable in a single snapshot (in Fig 7(a3)) and the task map was extracted through a timeseries of 165 snapshots; 2) The reconstructed δχ data yielded a higher task extraction performance than the reconstructed full χ data (in terms of *max*(*tcorr*) in (c2,c3)); 3) The δχ-depicted task activation patterns consists of spatially separated positive and negative blobs (bidirectional responses), which is different from the magnitude-depicted the prevailing positive response (cf. (b1, b3)); and 4) The low-resolution (2mm) χ image (a2) hinders the cortical details (gyri and sulci) for the function/structure co-localization in comparison with the high-resolution (0.5mm) χ image (the background image in Fig 6(b2)). Overall, this 3T task fMRI experiment shows that our BOLD perturbation model is also applicable to low-resolution low-field individual fMRI data analysis.

## Discussion

Under linear approximations of T2* phase imaging and with the additive BOLD perturbation model, we can trace the BOLD activity (in a perturbation term) at different imaging stages. Specifically, BOLD activity is expressed by δχ in the original source (with a χ + δχ model), δ*b* in the fieldmap (*b* + δ*b* model), and δ*P* in the T2* phase image (*P* + δ*P* model). Reversely, the BOLD signal decomposition enables inverse mappings of pure BOLD responses in the magnetic source dataspace by solving an inverse fMRI problem while excluding the static non-BOLD signals.

In our previous publications [5,16,20,39], we have reported on a two-step forward mapping model for brain MRI and a corresponding two-step inverse mapping model (a linear CIMRI model [20]) that is for χ source reconstruction (called χ tomography in the context of source reconstruction in medical imaging [16,30,31]). We apply the two-step inverse mapping model for brain full χ source reconstruction, as illustrated by a data flow *P*[**r**,t]→ *b*[**r**,t] → χ[**r**,t]. With the BOLD perturbation model, we are allowed to trace the data transformations on a BOLD signal during BOLD fMRI data acquisition, as illustrated by δχ(**r**,t)→ δ*b*(**r**,t) → δ*P*[**r**,t]. Correspondingly, by rendering inverse mappings, as denoted by δ*P*[**r**,t] → δ*b*[**r**,t] → δχ[**r**,t], we reproduce the original intrinsic pure BOLD magnetic responses. Both χ and δχ reconstructions are implemented by CIMRI, which is essentially a linear inverse imaging solution.

The reconstruction of 4D χ[**r**,t] dataset from a 4D *P*[**r**,t] dataset (unwrapped phase images) is essentially a repetition of quantitative susceptibility mapping (QSM) [34–38] at a timeseries of snapshot images. There is an emergence of brain functional mapping in the reconstructed magnetic susceptibility source data space, as termed by functional χ mapping [16] or functional QSM [43,44], for more direct sourced-based brain function depiction. In fact, the QSM was demonstrated in Fig 3 with a signal snapshot volume reconstruction. In the framework of QSM, one difficulty is to unwrap the severely wrapped phase images and remove the phase background, which can be effectively and efficiently solved through the use of Laplacian unwrapping technique [27,28] (in Eq (8)). Fortunately, the 4D δχ[**r**,t] reconstruction does not involve the awkward phase unwrapping and phase background removal at all. The other difficulty is a 3D dipole inversion, which is afflicted by a “divide-by-zero” problem, for which we approach through a TVB iteration technique by dealing with a “multiply-by-zero” problem instead [20,30,34,39,40].

A BOLD activity map can be extracted from a timeseries of T2* magnitude images through the use of SPM (http://www.fil.ion.ucl.ac.uk/spm/), which is a software tool for whole brain functional mapping. In our high-field high-resolution experiment by a GRE-EPI sequence, we could cover a chunk of brain superior portion (with a thickness of 28.8mm from brain top), which is not suitable for whole brain functional mapping by SPM. Expediently, we presented the *tcorr* maps for functional mappings.

Our previous reports [5,17] show that the magnitude-based brain fMRI suffers from nonlinear distortional mappings. With the BOLD perturbation model, we herein show that there is a nonlinear interaction between the dynamic BOLD perturbation and the static background in a T2* magnitude signal that manifests as a quadratic nonlinearity under the 1^st^ order Taylor expansion (in Appendix). This implies that a BOLD activity cannot be separated from the non-BOLD background in a T2* magnitude signal. A high field (or a long T_E_) aggravates the nonlinear magnitude signal coupling due to more nonlinear terms introduced from high-order Taylor expansions. This poses a caveat on the magnitude-based brain fMRI and neuroimaging from the viewpoint of inseparable magnitude signals[17]. In comparison, we can derive a linear T2* phase imaging model from the 1^st^-order Taylor expansion. It is understandable that higher-order Taylor expansions will bring more phase nonlinearity [7].

The Taylor expansion on a preliminary proton precession signal (in Appendix) is a mathematic manipulation in a small phase angle condition, |γ*b*T_E_| << 1 rad, which is seldom satisfied in a practical BOLD fMRI experiment. Nevertheless, the Taylor expansion reveals different magnitude and phase nonlinear behaviors [7]. Theoretically, the phase perturbation term δ*P* (extracted by complex division) could better meet the small phase angle condition (see Fig 4(a1,a2) for |δ*P*| < 1 rad) than the full phase signals (see Fig 5(b1) for *P*∈(-6, 6) rad). That is, the δχ reconstruction suffers less nonlinearity than the χ reconstruction. It is remind of the displays of {*P*, δ*P*, χ, δχ} in different window levels (grayscales or colorbars). In the sense of depiction accuracy, we advocate brain functional χ mapping in the reconstructed δχ dataspace instead of χ dataspace.

The brain χ source may assume a bipolar-valued distribution in reflection of the brain tissue diamagnetism and paramagnetism. The χ-expressed brain structure reconstruction from a snapshot T2* phase volume is essentially a QSM technique. It is a new concept to represent a brain tissue structure image by a bipolar-valued χ map [45], which is only available by a computational imaging approach that allows negative values. Indeed, there exist both χ-expressed positive and negative brain tissues. Specifically, a reconstructed brain χ image may assume negative values (e.g. χ_water_ < 0) and positive values (e.g. χ_ferritin_ > 0 and χ_myelin_ > 0), and the BOLD δχ perturbation may assume negative values (e.g., χ_oxyHb_ < 0) and positive values (e.g. χ_deoxyHb_ > 0). The linear T2* phase imaging retains the signs of bipolar-valued χ source, whereas the T2* magnitude imaging completely suppresses the signs due to nonnegative mapping. In particular, our experiment reveals concurrent positive and negative BOLD χ responses (see δχ_tcorr_ in Figs 5 and 6), which may indicate the biological antagonism and homeostasis during a brain activity: a stimulus evokes a positive response in one region (excitation) meanwhile a negative response in another region (inhibition). In comparison, the conventional magnitude-depicted *fmap* reveals prevailing positive responses over the brain (see *A*_tcorr_).

In this report, we demonstrated our brain functional BOLD perturbation model for task fMRI data analysis through the use of two single-subject task fMRI datasets acquired in different experiment settings. Both experimental data analyses produced similar results. Since BOLD changes are relatively small, we cannot observe a brain function activity from a snapshot image. Through a designed task paradigm, we can infer the brain activity from a timeseries of repeating measurements through a task correlation method. Specifically, we used a timeseries of 50 snapshots (in 5cylces) in our 7T task fMRI experiment and a longer timeseries of 165 snapshots (in $5\frac{1}{2}$ cycles) in our 3T experiments. Besides the individual fMRI study, the brain functional BOLD perturbation model is in principle applicable to group-level multi-subject brain function analysis. This is an ongoing research topic.

The brain functional BOLD perturbation model is very useful for brain fMRI data analysis and computationally inverse mapping for brain χ source reconstruction. It is also useful for numerical BOLD fMRI simulations [14,46]. In principle, a BOLD fMRI signal is, in general, nonlinearly generated from a magnetic source (primarily χ); consequently, neither the magnitude nor the phase signal is a faithful representation of the internal magnetic source. The BOLD perturbation model includes a linearization strategy: extracting a weak dynamic signal from a static background-dominated nonlinear signal for linear dynamic signal processing. Specifically, we separate a small BOLD response in a part of fMRI phase by complex division and reconstruct the pure BOLD χ response by inverse mapping under linear approximations, thereby avoiding the inherent fMRI nonlinearity [7].

## Summary and conclusion

Brain activity only contributes to a very small portion of a brain fMRI signal (T2* magnitude signal), which has been modeled by a BOLD contrast mechanism. We propose using a BOLD perturbation model for better understanding the BOLD fMRI model from the viewpoint of MRI transformations. The BOLD perturbation model originally represents a neurovascular activity by a small additive perturbation term (δχ) in a magnetic-susceptibility-expressed state (χ), i.e., separating a weak dynamic BOLD activity from an overwhelming static background in the expression of χ source. Under linear approximations of tissue magnetization, the BOLD activity is represented in a δχ-induced fieldmap perturbation (δ*b*). Finally, T2* imaging conveys a BOLD activity in a complex T2* dataset. By performing a complex division on the timeseries of T2* phase images (usually wrapped), we can extract a temporal phase change (relative to a baseline) that is construed as the BOLD phase perturbation (δ*P*). Under linear approximations of T2* phase imaging, we show in theory that the BOLD perturbation model leads to a cascade of linear mappings: δχ→δ*b*→δ*P*. By inverse mappings (δ*P*→δ*b*→δχ), we reconstruct the BOLD δχ source from the BOLD δ*P* image. For the task-evoked BOLD fMRI experiment, we extracted the brain functional activity map from the reconstructed BOLD δχ data in a measure of task correlative response. In this proof-of-concept experiment, we demonstrated the BOLD perturbation model for brain functional data analysis and found the bidirectional brain χ responses in the reconstructed magnetic source dataspace. We also show that high-field high-resolution data enable more informative function and structure depiction, especially the function/structure association visualization with rich cortical details.

In conclusion, we propose a BOLD perturbation model to represent the magnetic source as a dynamic BOLD response imposed on a static background, and thereby trace the components separately in the forward fMRI for data acquisition and the inverse mapping for BOLD χ source reconstruction. The reconstructed pure BOLD χ source (δχ) allows us to look into brain functional activity more directly (i.e., in magnetic χ expression) and more accurately (i.e., basically free from MRI-introduced transformations).

## Appendix

### A. Taylor expansion of single proton precession signal

A T2* voxel signal is a spatial average of numerous preliminary nuclear proton precession signals in a voxel space. Over a fieldmap *b*(**r**), a nuclear proton precession signal is given by exp(*iγT*_*E*_*b*(**r**)). The 1^st^ and 2^nd^ order Tayler expansions of the complex signal with respect to *b*(**r**) are

$$\begin{array}{l} \text{exp}\left(i\gamma T_{E}b)\right)=\text{exp}\left(i\gamma T_{E}b\right)\\ =1+i\gamma bT_{E}+\frac{{\left(i\gamma bT_{E}\right)}^{2}}{2!}+\frac{{\left(i\gamma bT_{E}\right)}^{3}}{3!}+\cdots \\ \approx 1+i\gamma bT_{E}+\frac{{\left(i\gamma bT_{E}\right)}^{2}}{2!}\;\;\;\;\;\;\;\;\;{(\text{2}}^{\text{nd}}\text{-order}\;\text{approx}.)\\ \approx 1+i\gamma bT_{E}\;\;\;\;\;\;\;\;\;\;\;\;\;\;\;\;\;\;\;\;\;\;\;\;\;\;\;\;\;\;\;\;\;\;{(\text{1}}^{\text{st}}\text{-order}\;\text{approx}.) \end{array}$$ (A1)

Specifically, the 1^st^–order magnitude approximation is

$$\begin{array}{l} a(r)\approx \sqrt{1+{\left(\gamma \cdot b(r)\cdot T_{E}\right)}^{2}}\;\;\;\;\;\;\;\;\;(\text{1st}\;\text{Taylor}\;\text{expansion})\\ \overset{\sqrt{1+x}\approx 1+x/2}{\approx }1+{\left(\gamma \cdot b(r)\cdot T_{E}\right)}^{2}/2\;(\text{s}.\text{t}.|\gamma \cdot \delta b(r)\cdot T_{E}|<<1) \end{array}$$ (A2)

And the 1^st^-order phase approximation is

$$\begin{array}{l} p(r)\approx \text{arctan}\left(1+i\gamma bT_{E}\right){(1}^{\text{st}}\;\text{Taylor}\;\text{expansion})\;\\ =\text{arctan}\left(\gamma bT_{E}\right)\\ \overset{\text{arctan}x\approx x}{\approx }\gamma bT_{E}\;(\text{s}.\text{t}.|\gamma \cdot b\cdot T_{E}|<<1)\; \end{array}$$ (A3)

It is seen that the preliminary proton spin precession signal is of an inherent trigonometric nonlinearity. Its magnitude signal is of nonlinearity and non-negativity at all circumstances (taking on a quadratic behavior as the least nonlinearity at the 1^st^–order approximation). Its phase signal reveals linear behavior under linear approximations (resulting from the 1^st^ order Taylor approximation together with a trigonometric approximation: *arctan*(*x*) *≈ x*).

### B. Approximations of intravoxel dephasing magnitude and phase signals

A complex T2* signal is generated by an intravoxel dephasing average formula in Eq (5). The T2* magnitude and phase signals are calculated by

$$\left\{ \begin{array}{l} A[r,t]=\left|C[r,t]\right|=\frac{1}{\left|\Omega \right|}\sqrt{{\left(\sum_{r\prime \in \Omega }\text{cos}\left(\gamma b(r\prime ,t)T_{E}\right)\right)}^{2}+{\left(\sum_{r\prime \in \Omega }\text{sin}\left(\gamma b(r\prime ,t)T_{E}\right)\right)}^{2}}\;\;\\ P[r,t]=∠C[r,t]=\text{arctan}\left(\frac{\sum_{r\prime \in \Omega }\text{sin}\left(\gamma b(r\prime ,t)T_{E}\right)}{\sum_{r\prime \in \Omega }\text{cos}\left(\gamma b(r\prime ,t)T_{E}\right)}\right) \end{array}\right.$$ (B1)

Note the notation [**r**,t] for discrete voxel signals.

Based on the proton precession signal expansions in Appendix A, we have the 1^st^ and 2^nd^ order Taylor expansions of intravoxel dephasing signal as follows

$$\begin{array}{ll} C\text{[}r\text{,t]} & \approx \frac{1}{\left|\Omega \right|}\sum_{r\prime \in \Omega (r)}\left(1+i\gamma \cdot T_{E}\cdot b(r^{\prime },t)+\frac{{\left(i\gamma \cdot T_{E}\cdot b(r^{\prime },t)\right)}^{2}}{2!}\right)\;\;\;\;\;\;({\text{2}}^{\text{nd}}\;\text{Taylor}\;\text{expansion})\\ & \approx \frac{1}{\left|\Omega \right|}\sum_{r\prime \in \Omega (r)}\left(1+i\gamma \cdot T_{E}\cdot b(r^{\prime },t)\right)\;\;\;\;\;\;\;\;\;\;\;\;\;\;\;\;\;\;\;\;\;\;\;\;\;\;\;\;\;\;\;\;\;\;\;\;\;\;\;\;\;\;\;\;\;\;\;\;\;({\text{1}}^{\text{st}}\;\text{Taylor}\;\text{expansion}) \end{array}$$ (B2)

From (B2), we obtain the T2* magnitude signal at the 1^st^ order approximation

$$\begin{array}{ll} A^{raw}[r,t] & \approx \frac{1}{\left|\Omega \right|}\sum_{r\prime \in \Omega (r)}\left(1+\frac{{\left(\gamma \cdot b(r^{\prime },t)\cdot T_{E}\right)}^{2}}{2}\right)\;\;\;\;\;\;{(\text{1}}^{\text{st}}\;\text{Taylor}\;\text{expansion})\\ & =1+\frac{{\left(\gamma T_{E}\right)}^{2}}{2|\Omega |}\sum_{r\prime \in \Omega (r)}{\left(b(r^{\prime },t)\right)}^{2}\;\;\;\;\;\;\;\;\;\;\;\;\;\;\;\;\;(\text{quadratic}\;\text{nonlinearity}) \end{array}$$ (B3)

That is, in the least nonlinearity approximation at 1^st^ order Taylor expansion, a T2* magnitude image exhibits a quadratic nonlinearity. Therefore, we conclude that the T2* magnitude signal is a nonlinear mapping of the fieldmap in all circumstances. In particular, the quadratic nonlinearity causes the magnitude non-negativity, which prevents a static/dynamic decomposition as illustrated by the inseparability of δb and b_0_ in (δ*b*+*b*_0_)^2^ = (δ*b*)^2^+(*b*_0_)^2^+2·δ*b*·*b*_0_. Furthermore, T2* magnitude imaging is irreversible (for *b*(**r**) reconstruction) due to a nonlinear mapping like |±1| = 1.

Meanwhile, the 1^st^ and 2^nd^ approximations of T2* phase signal (from (B2)) are given by

$$\begin{array}{ll} P[r,t] & \approx \text{arctan}\left(\frac{\gamma T_{E}b[r,t]}{1-{\left(\gamma T_{E}\right)}^{2}b^{2}[r,t]/2}\right)\;\;\;\;\;\;({\text{2}}^{\text{nd}}\text{-order}\;\text{approx}.)\\ & \approx \gamma T_{E}b[r,t]\;\;\;\;\;\;\;\;\;\;\;\;\;\;\;\;\;\;\;\;\;\;\;\;\;\;\;\;\;\;\;\;\;\;\;\;\;\;\;\;\;\;\;\;\;\;\;\;\;{(\text{1}}^{st}\text{-order}\;\text{approx}.)\\ \text{with} & b[r]=\frac{1}{\left|\Omega \right|}\sum_{r\prime \in \Omega (r)}b(r^{\prime })\;\;\;\;\;\;(\text{intravoxel}\;\text{fieldmap}\;\text{average})\\ & b^{2}[r,t]=\frac{1}{\left|\Omega \right|}\sum_{r\prime \in \Omega (r)}b^{2}(r^{\prime },t) \end{array}$$ (B4)

which shows that T2* phase signal is nonlinearly related to the fieldmap in a general setting (at 2^nd^ and higher expansions).

## Supporting information

S1 Fig T2* magnitude image slices in a snapshot volume at a time point t_1_ (captured by a GRE-EPI sequence). The z number (in units of mm) is the slice distance from brain top.
(TIF)

S2 Fig T2* phase image slices in a snapshot at a time point t_1_ (captured by a GRE-EPI sequence). (TIF)

S3 Fig Brain full χ source slices in a reconstructed 3D χ[x,y,z,t_1_] (a 3D χ reconstruction from Laplacian-unwrapped phase volume by CIMRI). (TIF)

S4 Fig Brain BOLD δχ source slices in a reconstructed 3D δχ[x,y,z,t_1_] (a 3D δχ reconstruction from a 3D δP by CIMRI). (TIF)

S5 Fig Task-correlated 3D brain functional map from the EPI magnitude dataset. The 3D *tcorr fmap* was converted into z-scores.
(TIF)

S6 Fig Task-correlated χ-depicted 3D brain functional map from a reconstructed 4D χ dataset (including brain background χ_0_). The 3D *tcorr fmap* was converted into z-scores.
(TIF)

S7 Fig Task-correlated δχ-depicted 3D brain functional map from a reconstructed 4D δχ dataset (excluding brain background χ_0_). The 3D *tcorr fmap* was converted into z-scores.
(TIF)

S8 Fig A χ-depicted brain cortex structure. Multiple axial slices from a reconstructed 3D χ volume (image enhanced).
(TIF)

S1 Data S1Data.rar (compressed raw 7T magnitude, part 1 of 2 parts). (RAR)

S2 Data S2Data.rar (compressed raw 7T magnitude, part 2 of 2 parts). (RAR)

S3 Data S3Data.rar (compressed raw 7T phase, part 1 of 2 parts). (RAR)

S4 Data S4Data.rar (compressed raw 7T phase, part 2 of 2 parts). (RAR)

S5 Data S5Data.rar (compressed raw 3T magnitude, part 1 of 3 parts). (RAR)

S6 Data S6Data.rar (compressed raw 3T magnitude, part 2 of 3 parts). (RAR)

S7 Data S7Data.rar (compressed raw 3T magnitude, part 3 of 3 parts). (RAR)

S8 Data S8Data.rar (compressed raw 3T phase, part 1 of 3 parts). (RAR)

S9 Data S9Data.rar (compressed raw 3T phase, part 2 of 3 parts). (RAR)

S10 Data S10Data.rar (compressed raw 3T phase, part 3 of 3 parts). (RAR)

## Abbreviations

BOLD: blood oxygenation level dependence
CIMRI: computed inverse magnetic resonance imaging
EPI: echo-planar imaging
*fmap*: functional map
fMRI: functional magnetic resonance imaging
GRE: gradient-recalled echo
MRI: magnetic resonance imaging
QSM: quantitative susceptibility mapping
*tcorr*: temporal correlation
