# Supplementary figures and images for: Brain functional BOLD perturbation modelling for forward fMRI and inverse mapping
Source: PLoS One. 2018 Jan 19;13(1):e0191266. doi: 10.1371/journal.pone.0191266 (PMC5774799; doi:10.1371/journal.pone.0191266)

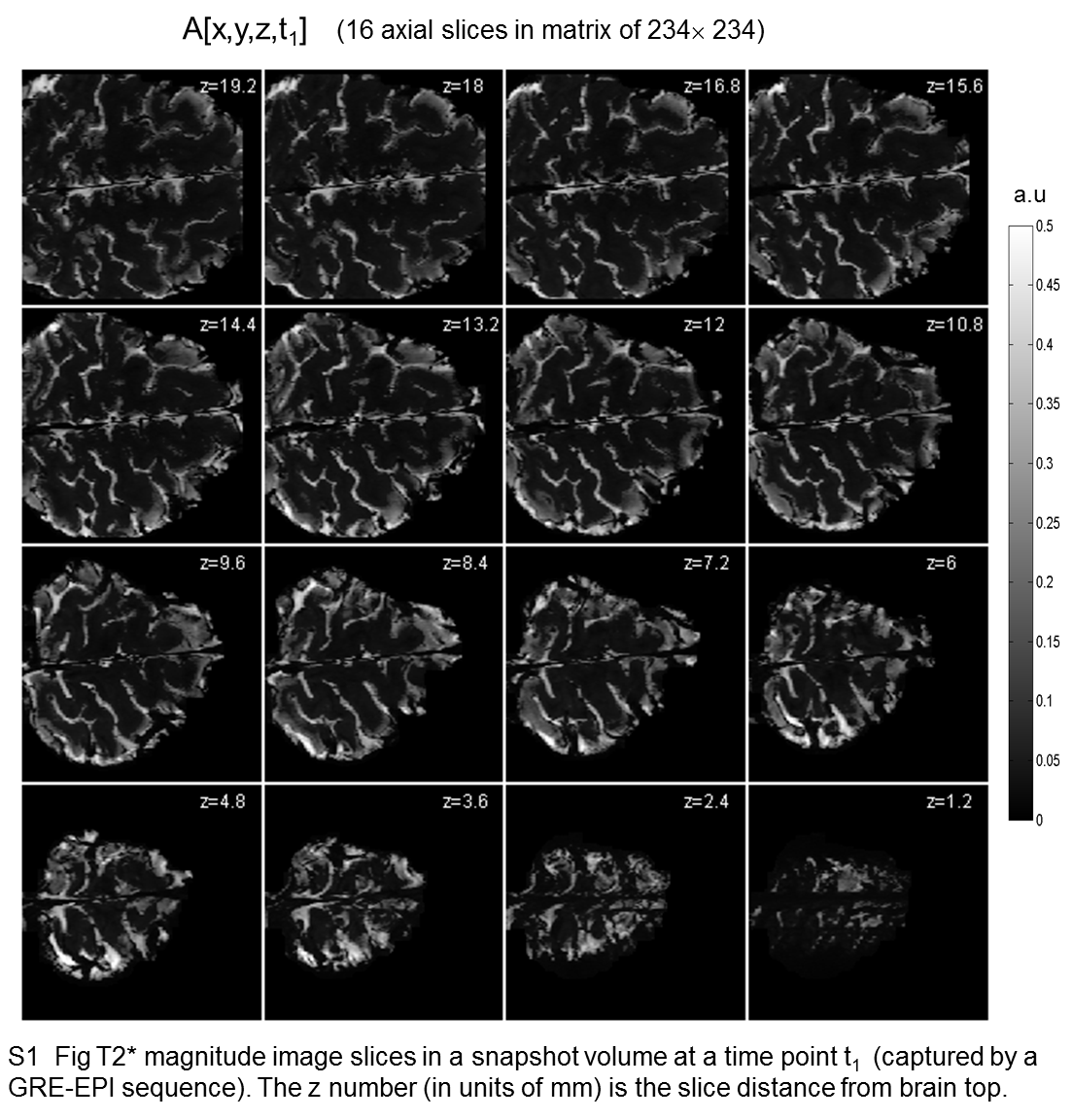

Supplement: S1 Fig — The z number (in units of mm) is the slice distance from brain top. (TIF) [file pone.0191266.s001.tif]

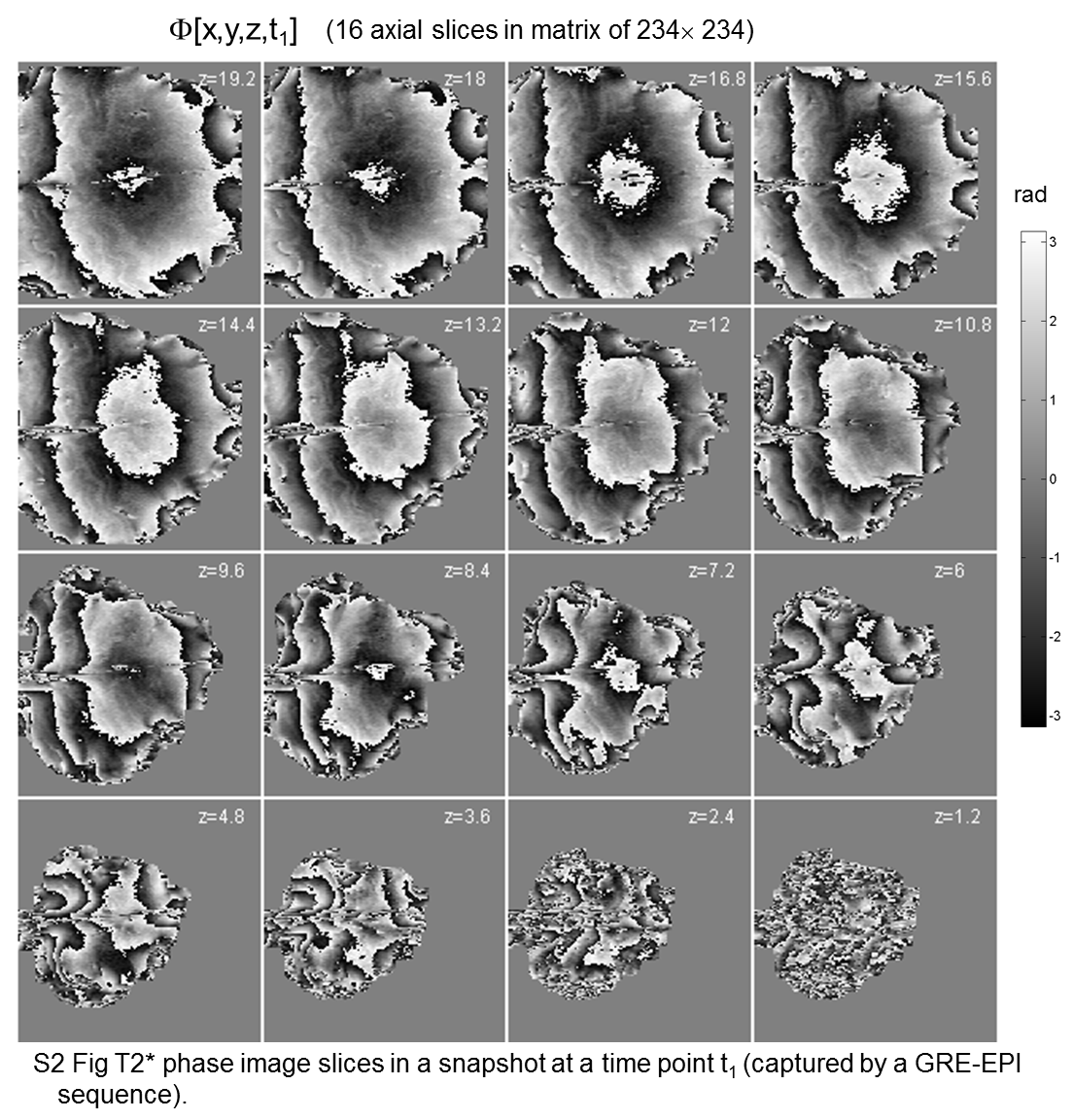

Supplement: S2 Fig — (TIF) [file pone.0191266.s002.tif]

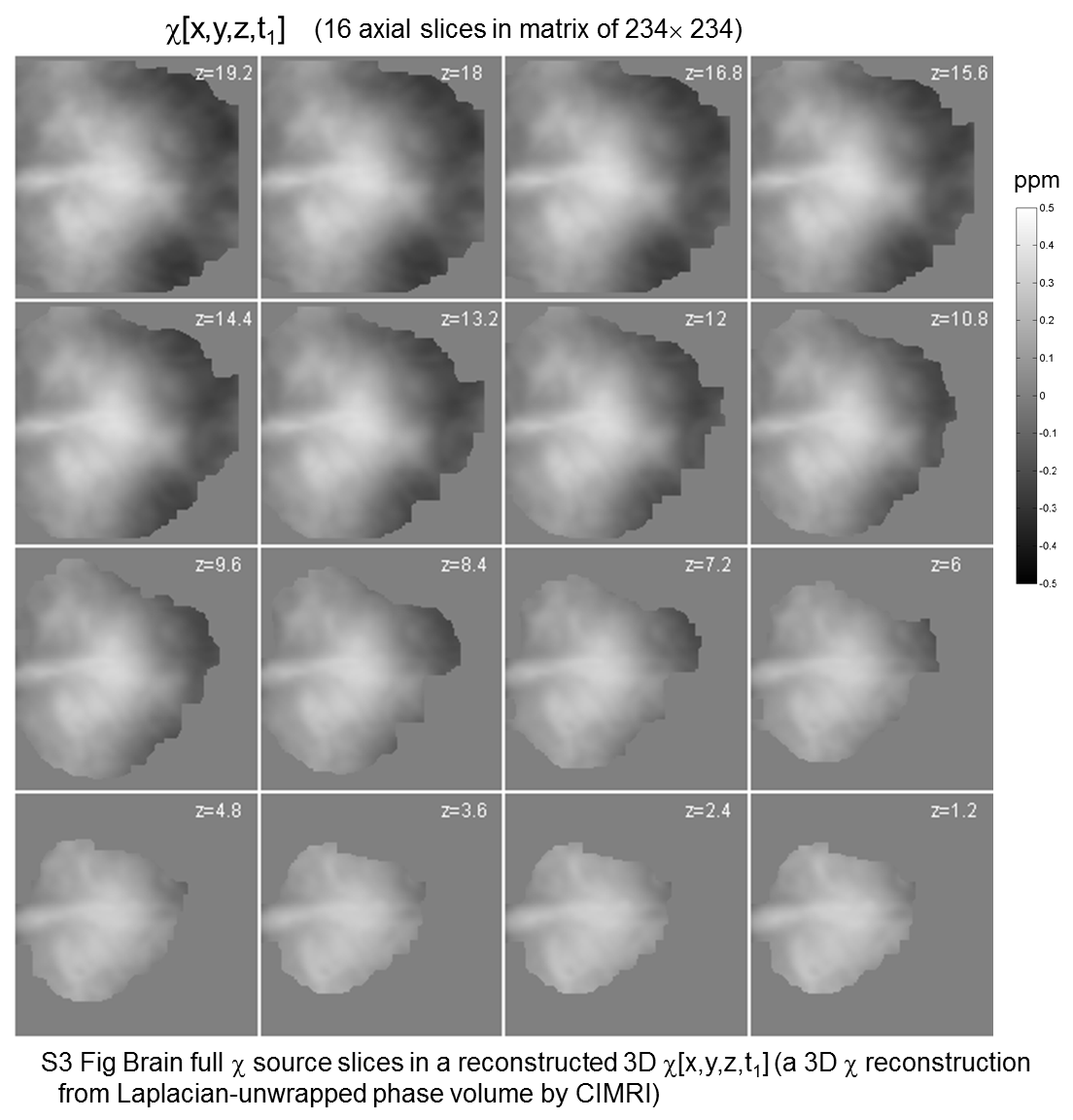

Supplement: S3 Fig — (TIF) [file pone.0191266.s003.tif]

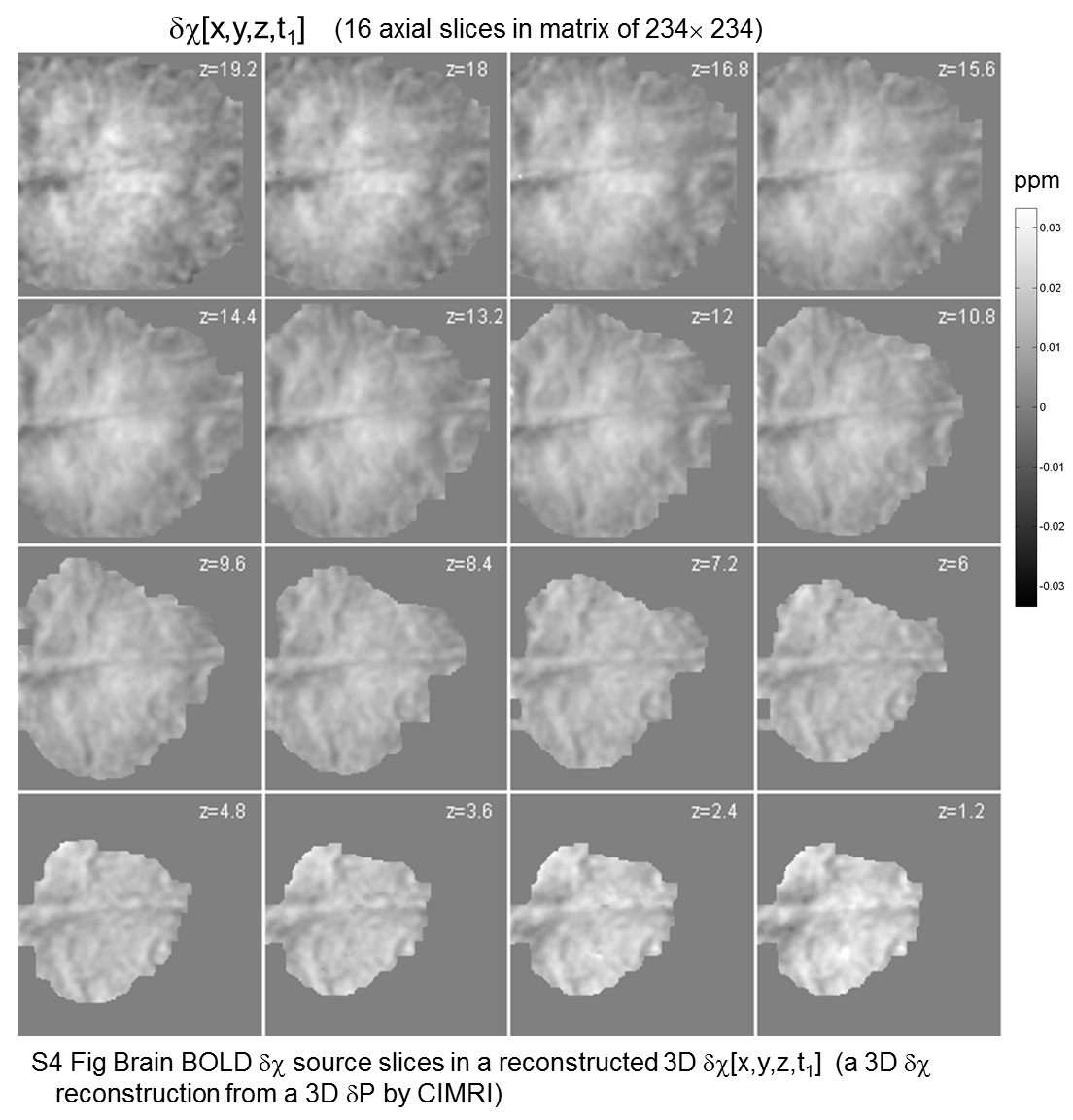

Supplement: S4 Fig — (TIF) [file pone.0191266.s004.tif]

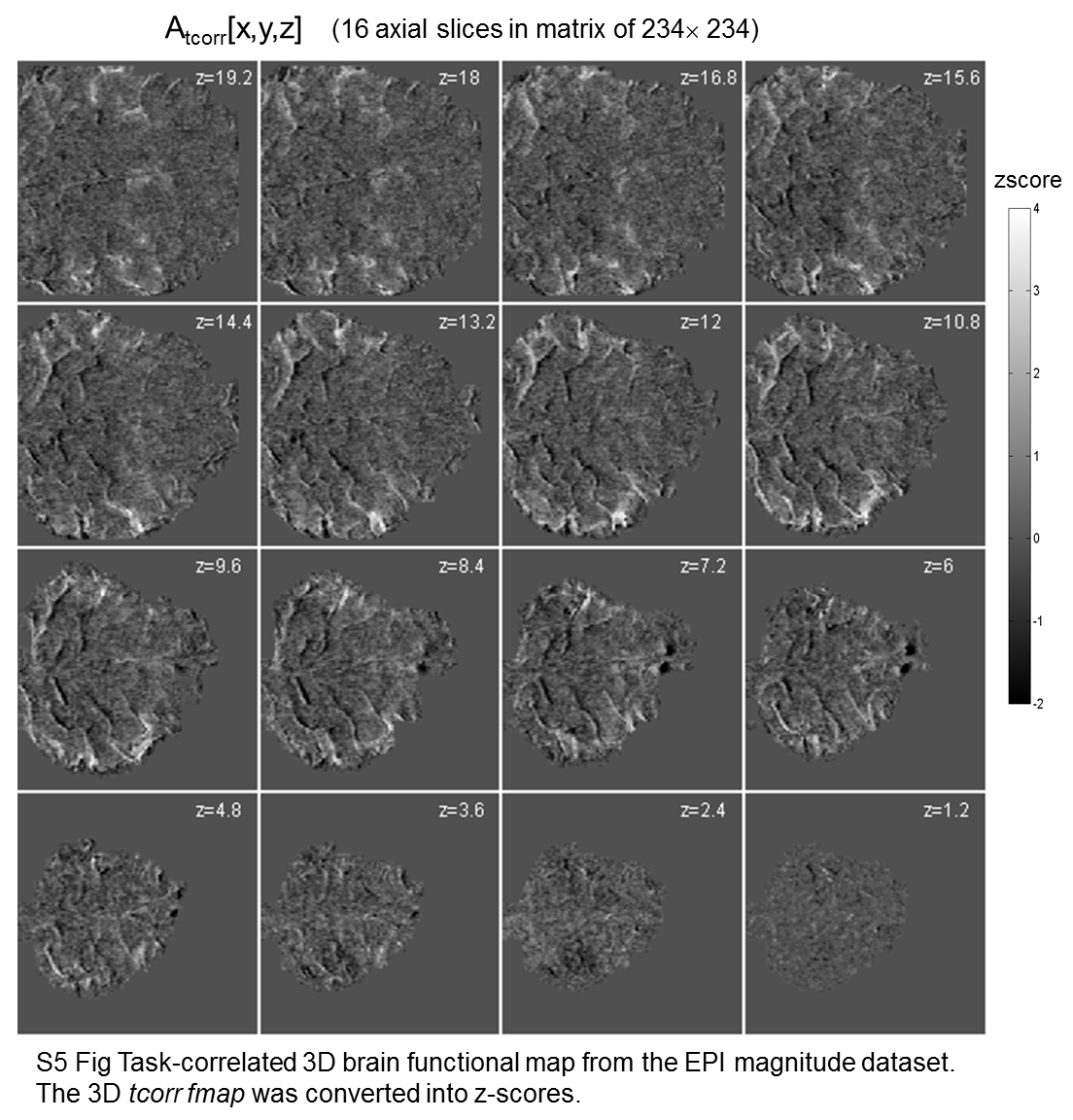

Supplement: S5 Fig — The 3D tcorr fmap was converted into z-scores. (TIF) [file pone.0191266.s005.tif]

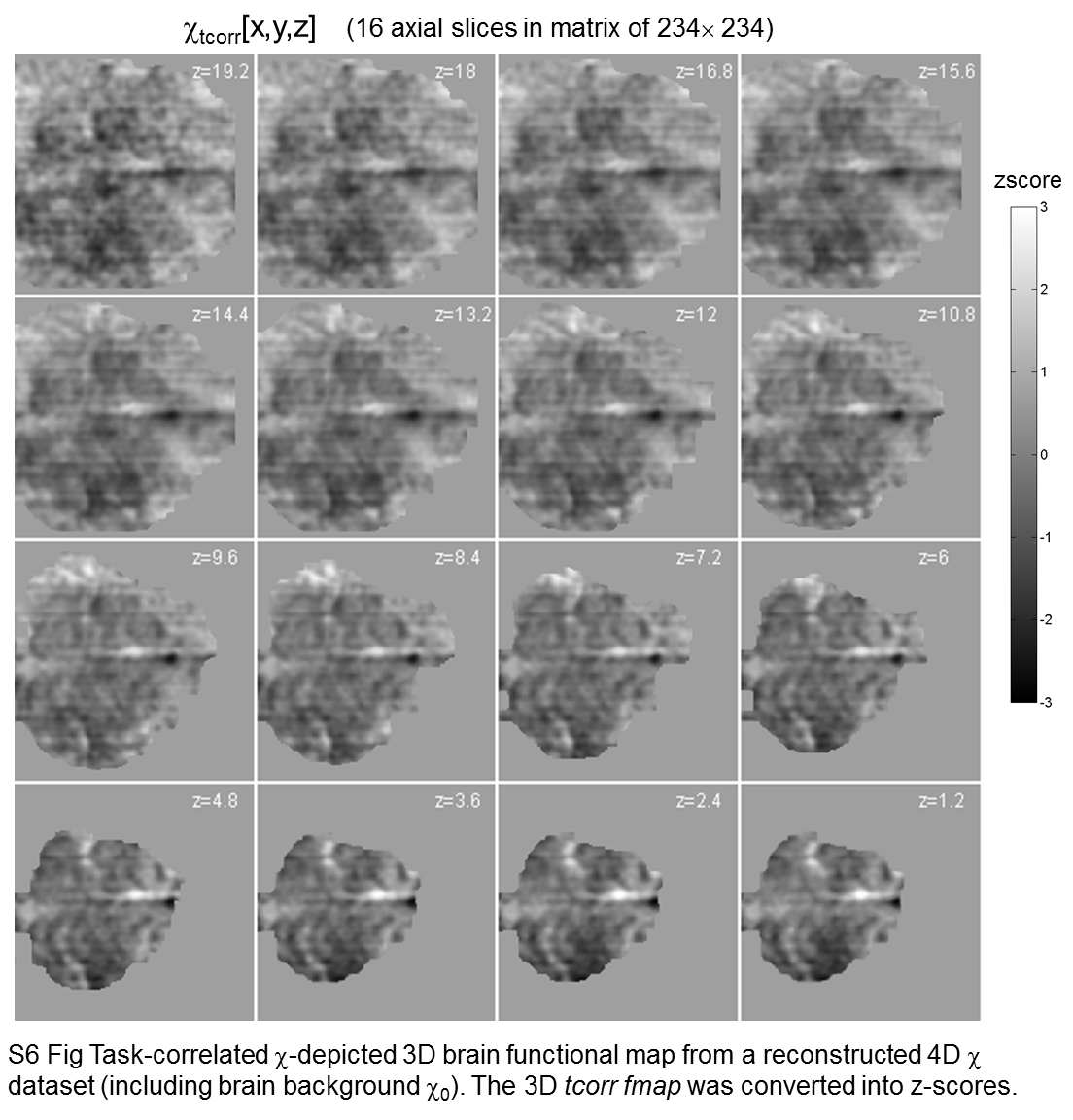

Supplement: S6 Fig — The 3D tcorr fmap was converted into z-scores. (TIF) [file pone.0191266.s006.tif]

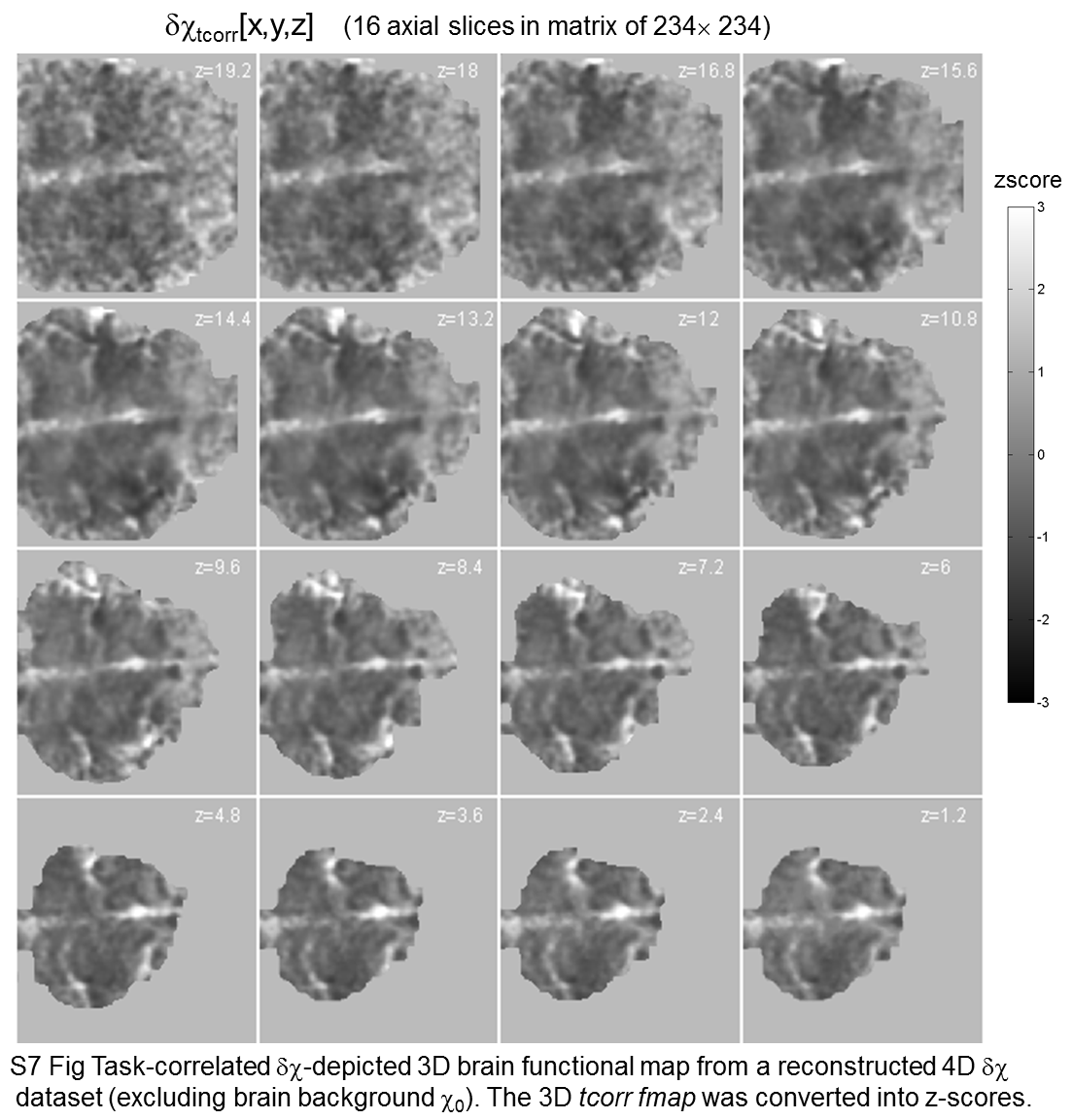

Supplement: S7 Fig — The 3D tcorr fmap was converted into z-scores. (TIF) [file pone.0191266.s007.tif]

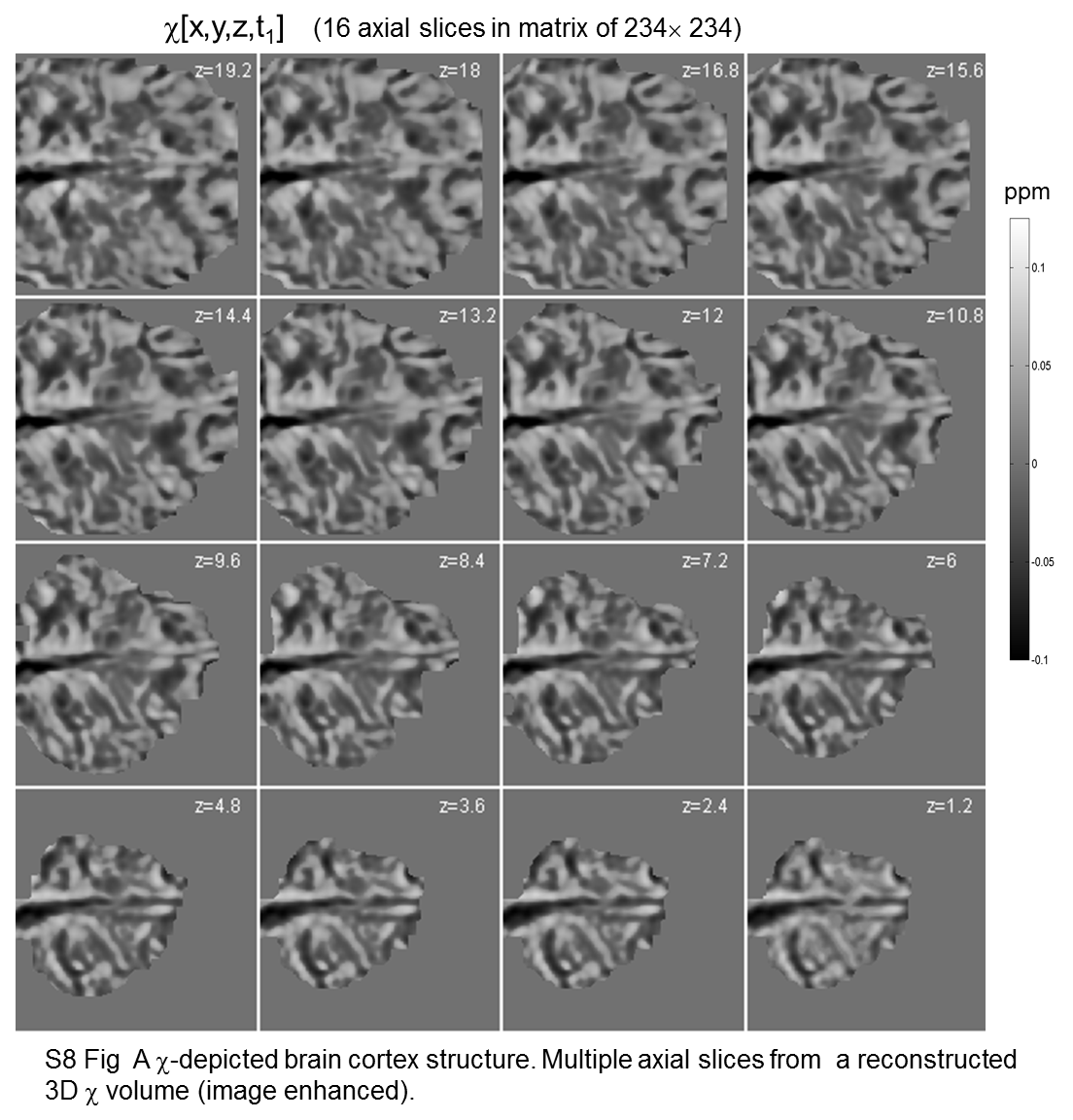

Supplement: S8 Fig — Multiple axial slices from a reconstructed 3D χ volume (image enhanced). (TIF) [file pone.0191266.s008.tif]
